# Co-expression of holin gene improves heterologous cellulase secretion and surface display by lactic acid bacteria *Lactococcus cremoris*

**DOI:** 10.1186/s13036-026-00625-0

**Published:** 2026-01-19

**Authors:** Petra Štravs, Lara Repar, Henri-Pierre Fierobe, Stéphanie Perret, Aleš Berlec

**Affiliations:** 1 Department of Biotechnology, Jožef Stefan Institute, Ljubljana, Slovenia; 2 Interdisciplinary Doctoral Study Program in Biosciences, University of Ljubljana, Ljubljana, Slovenia; 3 Aix-Marseille Université, CNRS, LCB-UMR7283, Marseille, France; 4 Faculty of Pharmacy, University of Ljubljana, Ljubljana, Slovenia

**Keywords:** *Lactococcus cremoris*, Holin, Endolysin, Cellulase, Secretion, Surface display

## Abstract

**Background:**

Genetically modified *Lactococcus cremoris* strains producing heterologous cellulases emerge as promising candidates for cost-effective bioprocessing of plant biowaste into high-value organic compounds. A key challenge in enabling efficient cellulose degradation by *L. cremoris* remains the effective secretion of heterologous cellulases. A potential solution would be to use genetic engineering to enhance the permeabilization of bacterial cell membrane and cell wall to facilitate the release of cellulases. This could be achieved by the co-expression of cellulase genes with genes encoding the prophage lytic proteins such as holin and endolysin.

**Results:**

Co-expression of genes encoding the prophage protein holin and heterologous cellulases Cel5I, Cel9A and Cel5H in *L. cremoris* significantly improved secretion of Cel5I (up to 15-fold) and Cel9A (at least 3-fold), as well as surface display of Cel5I, Cel5H and Cel9A. Comparable levels of secretion and surface display of cellulases were observed regardless of whether the holin gene was co-expressed from a plasmid or inserted into the genome. In contrast, co-expression of gene encoding the prophage protein endolysin did not improve secretion of cellulase even when produced simultaneously with its partner protein holin.

**Conclusions:**

Co-production of holin with heterologous cellulases appears as a promising strategy for improving cellulolytic capability of *L. cremoris* by increasing their secretion. This might advance the development of consolidated bioprocesses involving lactic acid bacteria for valorisation of cellulosic material. Additionally, the same approach might likewise be employed to enhance the secretion and surface display of other heterologous recombinant proteins.

**Supplementary Information:**

The online version contains supplementary material available at 10.1186/s13036-026-00625-0.

## Background

*Lactococcus cremoris* is one of the best characterized species of lactic acid bacteria (LAB). It has a long history of safe use in food and has therefore acquired status GRAS (Generally Regarded As Safe) [1]. With a fully sequenced genome and well-developed genetic tools, *L. cremoris* represents a model organism for the genetic engineering of LAB [1, 2]. Furthermore, *L. cremoris* has proven to be a promising host organism for the expression of recombinant proteins, and has been genetically engineered for a variety of applications in pharmaceutical biotechnology and the food industry. It offers several advantages over conventional bacterial system *Escherichia coli*, including the absence of endotoxins, a small genome, the absence of inclusion body formation and only one identified extracellular housekeeping proteinase [1]. *L. cremoris* is a homofermentative producer of the highly demanded and widely used lactic acid that serves as pH regulator, solvent, preservative, release agent and building block for the production of biodegradable plastics. It is therefore an interesting candidate for a more economical consolidated production of lactic acid from biowaste plant biomass [1, 3]. The primary constituent of plant biowaste is cellulose [4]. In order for bacteria to use the cellulose as a carbon source, they must produce cellulases that degrade the cellulose, as well as efficient transport systems that enable the import and utilization of cellulose degradation products [5]. The *L. cremoris* strain NZ9000 already contains an active cellobiose-specific phosphotransferase transporter that imports cellobiose [6], a major product of cellulose degradation by certain cellulases [7–9], into the cell. However, since *L. cremoris* possesses no endogenous cellulases, it is necessary to achieve efficient expression and secretion of heterologous cellulases in this organism in order to use it for cellulose transformations. Strong promoters for constitutive or inducible overexpression of heterologous proteins in *L. cremoris* have already been identified [10–12]. The major limiting factor in recombinant protein production by *L. cremoris* is efficient secretion of the overexpressed proteins, which also remains a challenge in development of cellulose-degrading LAB [5]. The native extracellular proteins of *L. cremoris* are secreted via the Sec pathway [13]. To target a recombinant protein to the Sec secretion machinery in *L. cremoris*, a specific signal peptide is fused to its N-terminus. Most commonly, the signal peptide of the only major secreted endogenous Usp45 protein [13] and its synthetic variants are used [14]. However, overexpression of recombinant proteins using a strong promoter often leads to saturation of the transmembrane transporters, which can also result in accumulation of misfolded proteins. Therefore, several strategies have been developed to improve the secretion yield of functional recombinant proteins in *L. cremoris*, such as insertion of short peptide sequences with negative net charge between the signal peptide and the mature protein [15, 16], inactivation of the extracellular housekeeping proteinase HtrA [17], co-expression of chaperones [18] and optimization of culture conditions [19]. Nevertheless, most of these approaches have not proven effective for all recombinant proteins.

Recently, a new approach based on the prophage protein holin was used in *L. cremoris* to improve the secretion yield of recombinant proteins staphylococcal nuclease and fimbrial adhesin FaeG [20]. Holin and its partner enzyme endolysin are lytic proteins that are involved in the prophage lytic cycle. Upon synthesis, holin is transported via the signal recognition particle (SRP) pathway to the cytoplasmic membrane, where it oligomerises to form pores [20, 21]. Very small pores cause depolarization of the membrane, while larger pores allow passage of endolysin to the cell wall. Endolysin cleaves bacterial peptidoglycan, leading to the degradation of the cell wall and the subsequent release of phages [21]. Two mechanisms have been proposed to explain how holin promotes the secretion of recombinant proteins in host cells. This can be achieved (i) by forming membrane pores that allow nonspecific release of cytoplasmic proteins into the extracellular space, and (ii) by promoting the expression of the SRP pathway proteins, thereby providing an alternative secretory pathway to Sect [20]. Since there are only minor variations in Sec and SRP signal peptide sequences, an overlap between the Sec and SRP pathways could occur [20]. In the present study, we investigated the ability of holin to enhance the secretion of recombinant cellulases from *L. cremoris*. Building on the strategy used in the study by Guo et al. [20], where holin alone was used to improve the secretion of target proteins, we overexpressed both holin (GenBank: ADJ61052.1) and endolysin (GenBank: ADJ61051.1) of the endogenous prophage, either alone or simultaneously, to improve the secretion of three different cellulases. Target cellulase and holin/endolysin were expressed from separate promoters, allowing their independent gene expression regulation. Furthermore, the gene encoding holin was integrated into the *L. cremoris* genome under an inducible promoter for more convenient use of the system. The inducing conditions for overexpression of holin either from plasmid or genome were optimized and evaluated for improved cellulase secretion. Besides, we also evaluated the effect of holin on surface display of cellulases containing surface anchor. Additionally, the impact of holin on cell viability and permeability of the cell membrane was evaluated.

## Methods

### Construction of expression plasmids

The sequences of primers (IDT) and descriptions of plasmids used in this work can be found in Supplementary material (Table S1). To prepare plasmids for expression of holin and endolysin (with or without protein tags), the corresponding genes were amplified from *L. cremoris* NZ9000 genome using suitable primers and inserted into the pNBBX plasmid between the restriction sites NcoI/XbaI [22]. In addition, the pNBBX plasmid encoding endolysin fused with N-terminal secretion signal peptide was prepared. The plasmid for polycistronic expression of both holin and endolysin with C-terminal protein tags was created by fusion of DNA fragments encoding holin and endolysin with asymmetric overlap extension PCR [23]. The fused fragment was inserted into the pNBBX plasmid between the restriction sites NheI/XhoI. The pNBBX plasmid was also used for the expression of cellulases Cel5I, Cel9A and Cel5H, as well as for the co-expression of cellulases with holin or/and endolysin. Detailed description of gene constructs and plasmid assembly is provided in Supplementary material. Cloning was carried out in *L. cremoris* NZ9000 strain, whereby the transformation was achieved with electroporation as described by Holo and Nes [24] using the BTX Gemini X2 electroporation system. For the isolation of the plasmids from *L. cremoris* cells, NucleoSpin Plasmid EasyPure (Macherey and Nagel) kit was used with an additional 30 min treatment with 62.5 U/mL mutanolysin (Sigma-Aldrich) and 195,000 U/mL lysozyme (Sigma-Aldrich). The accuracy of the newly constructed plasmids was confirmed by sequencing at Eurofins.

### Integration into the genome

To integrate the DNA cassette encoding the promoter PnisA, the holin gene with Myc tag at the 3’ end and the transcription terminator (Pnis_HolinM_TT) into *L. cremoris* NZ9000, we amplified this DNA fragment from the corresponding pNBBX plasmid. The amplicon was inserted into plasmid pMET306 [25] between the *Sal*I/*Kpn*I restriction sites, resulting in plasmid pMET306_Pnis_Holin_TT. This plasmid was prepared in a chemically competent *E. coli* strain Oneshot TOP10F’ (Invitrogen). The plasmid pMET306_Pnis_Holin_TT for integration at the tRNA-Ser locus (LLNZ_t13275) was introduced into *L. cremoris* NZ9000 by electroporation. To select the cells with integrated sequence, cells were plated on M17 agar medium supplemented with 5 g/L glucose (Formedium) and 5 µg/ml erythromycin (Sigma Aldrich). Integration of Pnis_Holin_TT sequence into the genome of *L. cremoris* NZ9000 strain was confirmed by sequencing of a genome fragment amplicon, obtained using primers Pmet306_For_G (aligning upstream of the tRNA-Ser locus (LLNZ_t13275) in the genome) and TT-R-Kpn aligning to the transcription terminator sequence of the Pnis_Holin_TT integrated construct. Sequencing was performed at Eurofins.

### Bacterial growth and gene expression

*L. cremoris* cells were grown at 30 °C in M17 medium (Millipore) enriched with 5 g/L glucose (Formedium) under microaerophilic conditions without agitation, while growth of *E. coli* was carried out at 37 °C in lysogeny broth medium under aerophilic conditions with agitation at 190 rpm. To retain the selection pressure in *L. cremoris* and *E. coli*, the medium was supplemented with chloramphenicol (Sigma-Aldrich) at a concentration of 10 µg/ml, or with erythromycin (Sigma Aldrich) at a concentration of 150 µg/ml, respectively. To induce gene expression in *L. cremoris* cells, the overnight culture was subcultured (1:50) into fresh medium. Expression of the holin and endolysin genes from the plasmid were induced at different time points (during early, mid or late exponential phase) and with different concentrations of nisin (Fluka; 5 ng/mL, 10 ng/mL and 25 ng/mL). In contrast, expression of holin gene integrated into the *L. cremoris* genome was induced at the time of inoculation or in the early exponential growth phase with 25 ng/mL or with 50 ng/mL nisin. Following induction, the bacterial cultures were incubated overnight.

### SDS-PAGE and western blotting

To confirm the production of holin and endolysin, cell lysates were prepared using a bead-beating method. The cell pellet harvested from a 10 mL overnight culture was resuspended in 400 µL of phosphate-buffered saline (PBS) and disrupted with 0.1 mm zirconia/silica beads using a bead beater (Precellys Evolution, Bertin) for three cycles of 1 min at 5,000 rpm. Between cycles, samples were incubated on ice. The beads were sedimented by quick centrifugation and the supernatant was combined with Laemmli sample buffer containing dithiothreitol (DTT) (Thermo Fisher Scientific) and heated for 10 min at 100 °C prior to electrophoresis. Concentrated or non-concentrated medium was used to evaluate cellulase secretion and the presence of recombinant endolysin in the overnight culture medium. To concentrate samples, the conditioned medium was collected and the proteins were precipitated using a final concentration of 10% trichloroacetic acid (TCA) (Sigma-Aldrich). Following precipitation, the pellets were rinsed with ice-cold acetone and resuspended in 5× Laemmli sample buffer. DTT was added prior to electrophoresis. In cases where the conditioned medium was not concentrated, Laemmli buffer supplemented with DTT was added directly. All samples were subsequently heated at 100 °C for 10 min. The proteins present in the concentrated conditioned medium were separated on a 10% Stain-Free gel (Bio-Rad), and the cell lysates were separated on a 12% Stain-Free gel at 35 mA. The sample loading of the cell lysates was normalized based on the optical density of the cell culture, while the same volumes of samples of the concentrated conditioned media were loaded onto the gel. After separation, the gel was photoactivated with UV for 1 min, and ChemiDoc MP Imaging System (Bio-Rad) and ImageLab software (version 5.1, Bio-Rad) were used for imaging. Precision Plus Protein™ All Blue Prestained Protein Standard (Bio-Rad) was used to determine the molecular weight of the target proteins.

Proteins were transferred from the gels to a nitrocellulose membrane (Bio-Rad) for 6 min using the Trans-Blot Turbo System (Bio-Rad). The membrane was blocked in 5% skim milk in Tris-buffered saline containing 0.05% Tween-20 (TBST) for 1 h at room temperature. Cellulases, holin and endolysin were detected by incubating the membrane overnight at 4 °C with specific antibodies in blocking buffer: cellulases with primary rabbit anti-flag antibody (DYKDDDDK tag Polyclonal antibody; Proteintech) diluted 1:10,000, holin with primary rabbit anti-myc antibody (MYC tag Polyclonal antibody; Proteintech) or primary mouse anti-myc antibody (MYC tag Monoclonal antibody; Proteintech) diluted 1:5,000, and endolysin with primary mouse anti-his antibody (6*His, His-Tag Monoclonal antibody; Proteintech) diluted 1:10,000. The membrane was then incubated for 1 h at room temperature with appropriate secondary antibodies: StarBright IgG Blue 520 fluorescent goat anti rabbit antibody (Bio-Rad) or StarBright IgG Blue 720 fluorescent goat anti mouse antibody (Bio-Rad); both diluted 1:10,000 in blocking buffer when used for cellulases and endolysin, and 1:5,000 when used for holin. The membrane was rinsed with 0.1%TBST three times following every incubation. ChemiDoc MP Imaging System (Bio-Rad) and ImageLab software (version 5.1, Bio-Rad) were used for image capture and processing. GelAnalyzer 19.1 software was used for gel analysis.

### Dot blot of bacterial cells

*L. cremoris* cultures were centrifuged (10 min, 6000 g, 4 °C), washed and resuspended in PBS to an OD_600_ of 4. The resulting cell suspensions (2 µL) were dropped onto a nitrocellulose membrane (Bio-Rad). Subsequent incubation with antibodies and membrane imaging were carried out as described above for Western blot.

### Activity of cellulases on amorphous cellulose

Phosphoric acid swollen cellulose (PASC) was used as a substrate to evaluate the activity of heterologously expressed cellulases that were either secreted or surface-displayed on *L. cremoris*. PASC was prepared from Avicel PH 101 microcrystalline cellulose according to the method of Wood [26]. Enzymatic activity was determined by measuring the soluble reducing sugars released into the supernatant using the ferricyanide method of Park and Johnson [27]. Sample preparation for secreted and surface-displayed cellulases followed the procedure of Štravs et al. [12]. Briefly for secreted enzymes, the culture supernatant was buffer-exchanged into 50 mM potassium phosphate (pH 7.0) and concentrated 20-fold using 10 kDa Vivaspin filters. The enzyme solution (150 µL) was incubated with 3 mL of substrate (3.5 g/L Avicel or PASC in 50 mM potassium phosphate, pH 7.0, containing 0.01% sodium azide) at 30 °C and 70 rpm for 24 h. For surface-displayed cellulases, *L. cremoris* NZ3900 cells were washed and resuspended in 50 mM potassium phosphate buffer (pH 7.0, 137 mM NaCl) to OD₆₀₀ of 28; 500 µL of this suspension was added to 3 mL substrate solution (4.0 g/L Avicel or PASC in 50 mM potassium phosphate, pH 7.0, 137 mM NaCl), yielding a final OD₆₀₀ of 4 with final concentration of sodium azide 0.01%. Cells were incubated with substrate for 24 h at 30 °C and 70 rpm. The amount of released sugars was expressed as glucose equivalents.

### Activity of cellulases on carboxymethyl cellulose

Cellulase activity was assessed on carboxymethyl cellulose (CMC) (Sigma-Aldrich) agar plates prepared with 1.5% agar and 0.5% CMC. Conditioned medium (5 µL) was spotted onto the plates and incubated overnight at 30 °C. The plates were then treated with 0.1% Congo red solution (Sigma-Aldrich) for 15 min, followed by washing with 1 M NaCl to visualize clearance zones as a result of enzymatic activity. The diameter of clearance zones was measured.

### Growth analysis

The growth of *L. cremoris* was monitored by measuring the optical density (OD) of cell suspensions in a microtiter plate at 30 °C for 24 h. OD values were recorded every 20 min at 595 nm using a Sunrise microplate reader (Tecan) with continuous shaking. Measurements were performed in three biological replicates, each with five technical replicates.

### Bacterial cell viability assay

To assess the viability of *L. cremoris* after expression of prophage lytic proteins, the number of colony-forming units per milliliter (CFU/mL) was determined. Expression of genes encoding prophage proteins was induced in exponential phase, followed by overnight cultivation. Then, the cultures were serially diluted tenfold in sterile PBS. Ten microliters from each dilution were dropped onto M17 agar plates supplemented with 5 g/L of glucose and incubated overnight at 30 °C.

### Determination of membrane-compromised cells

Overnight cultures with the optical density (OD_600_) of 1 were diluted in microplate with 15 µM propidium iodide solution (PI) (Molecular Probes) in ratio cells: PI (v/v) = 1: 1, to the final volume 200 µL. The suspension was resuspended and incubated at RT in the dark for 15 min. The fluorescence was measured in Infinite M1000 microplate reader (Tecan) with excitation at 485 nm and emission at 630 nm.

### Confocal microscopy

An aliquot of overnight cell culture (20 µL), adjusted to an OD_600_ of 4, was centrifuged (5 min, 5000 g, 4 °C), washed with 500 µL Tris-buffered saline (TBS), and resuspended in 300 µL of a 15 µM PI. The suspension was vortexed and incubated for 15 min at RT in the dark. For confocal microscopy, cells incubated in PI solution were centrifuged onto poly-L-lysin–coated slides (Sigma-Aldrich) using a StatSpin Cytofuge 2 cytocentrifuge (1000 g, 5 min, RT; Beckman Coulter). An LSM 710 confocal microscope (Carl Zeiss) was used to examine the prepared samples using excitation with a 543 nm laser. The emitted light was filtered through a 566–719 nm bandpass filter. All images that were included in the comparison were acquired under identical settings and analyzed using ImageJ software version 1.53k [28].

### Statistical analysis

Results that were subject to statistical analysis are expressed as means of three biological replicates ± standard deviation (SD), as specified. Statistical analyses were carried out in GraphPad (version 10.00) using ordinary one-way ANOVA with appropriate post hoc corrections. A p value < 0.05 was considered statistically significant.

## Results

### Inducible expression of recombinant lytic prophage proteins in *L. cremoris*

**Fig. 1:**
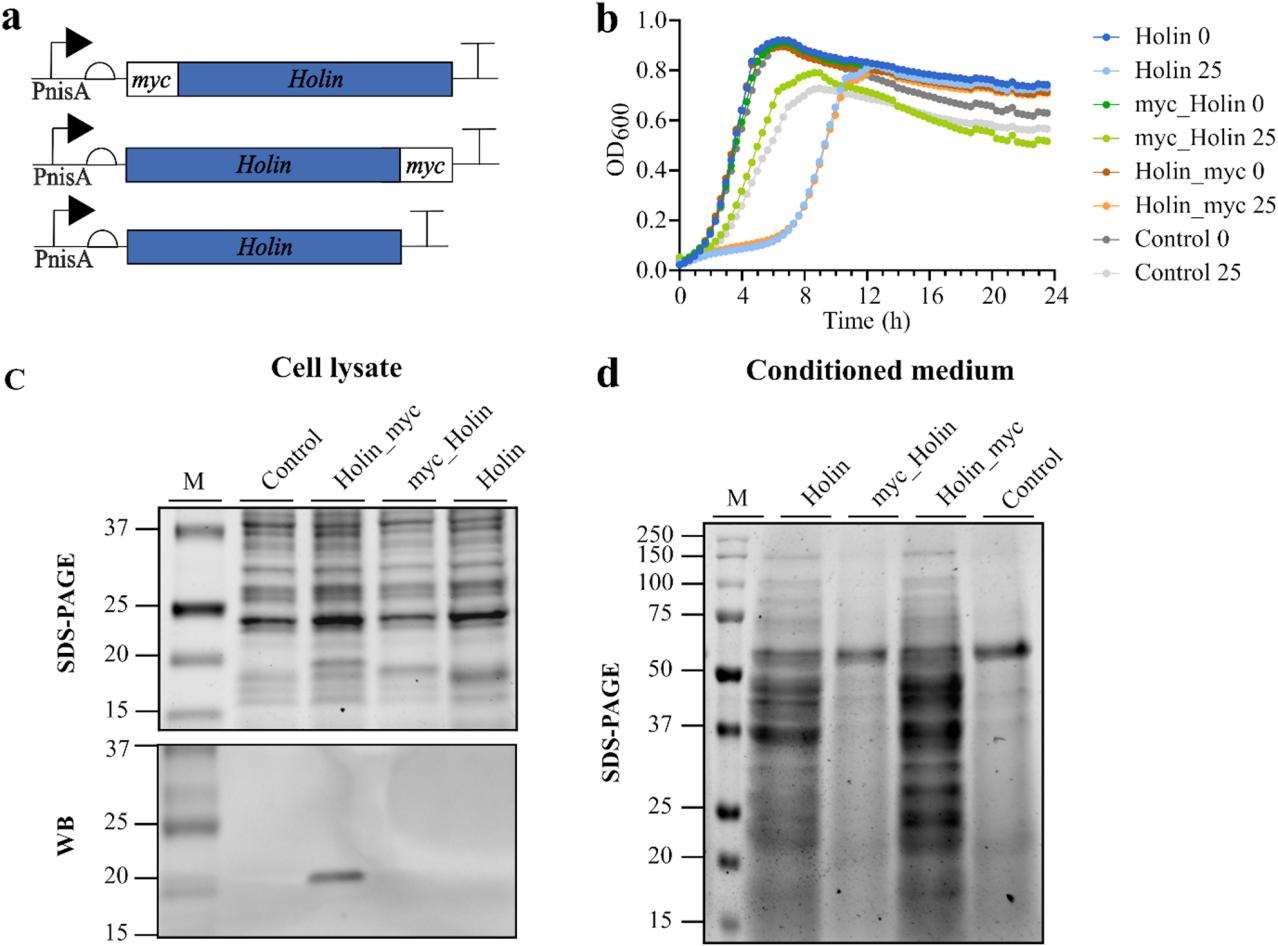
Holin gene expression in *L. cremoris*. (**a**) Genetic constructs designed for inducible expression of holin with or without the Myc tag (myc) at the N- or C-terminus of the protein. PnisA: nisin-inducible promoter. (**b**) Growth curves of *L. cremoris* strains transformed with empty pNBBX plasmid (Control) or with pNBBX plasmids containing the constructs for holin expression described in (**a**). Expression of holin gene was induced with 0 or 25 ng/mL nisin at the beginning of growth. Data are presented as mean of three biological replicates. (**c**) SDS-PAGE and corresponding Western blot of cell lysates and (**d**) SDS-PAGE of concentrated conditioned media of *L. cremoris* cells producing holin with or without the N- or C-terminal Myc-tag. Expression of holin gene was induced with 25 ng/mL nisin in the mid-exponential growth phase. In panels b-d, holin variants without or with the N- or C-terminal Myc tag are shown as holin, myc_holin and holin_myc, respectively. Control: *L. cremoris* strain transformed with empty pNBBX plasmid. M: molecular weight standard

**Fig. 2:**
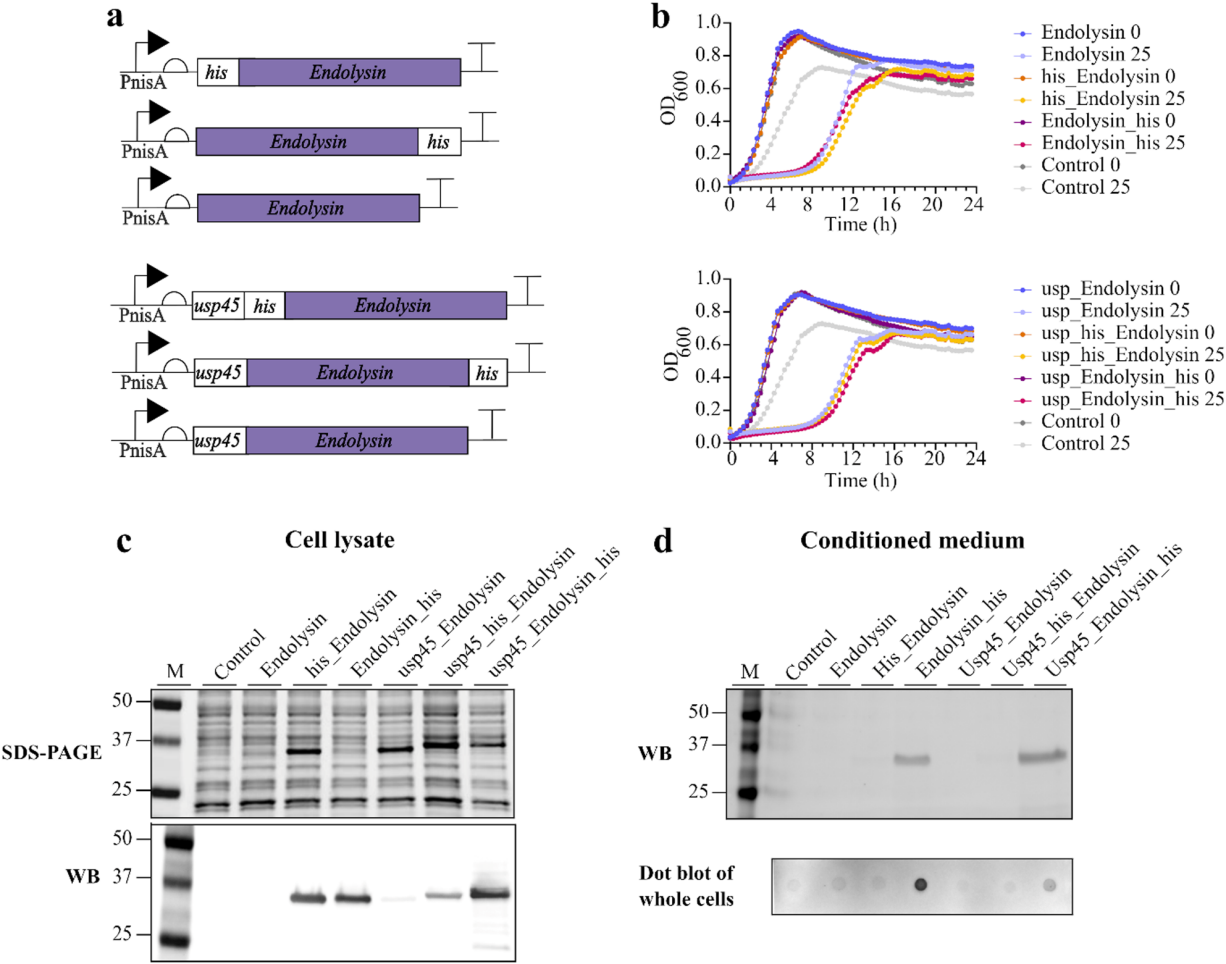
Endolysin gene expression in *L. cremoris*. (**a**) Constructs designed for inducible expression of endolysin with or without the His8 tag (his) at the N- or C-terminus and with or without the secretion signal peptide sequence (usp45). PnisA: nisin-inducible promoter. (**b**) Growth curves of *L. cremoris* strains transformed with empty pNBBX plasmid (control) or with plasmids containing the constructs for endolysin expression described in (**a**). Expression of endolysin was induced with 0 or 25 ng/mL nisin at the beginning of growth. Data are presented as mean of three biological replicates. (**c**) SDS-PAGE and corresponding Western blot of cell lysates, (**d**) WB of concentrated conditioned media and dot blot of whole *L. cremoris* cells expressing endolysin with or without the N- or C-terminal His8 tag. Endolysin expression was induced with 25 ng/mL nisin in the mid-exponential growth phase. In panels b-d, endolysin variants without or with the N- or C-terminal His8 tag are shown as endolysin, his_endolysin and endolysin_his, respectively. Endolysin variants with the N-terminal SP_Usp45_ signal sequence are denoted. Control: *L. cremoris* strain transformed with empty pNBBX plasmid. M: Molecular weight standard

First, the genes encoding the prophage proteins holin (GenBank: ADJ61052.1; 16.7 kDa) and endolysin (GenBank: ADJ61051.1; 31.7 kDa) were overexpressed in *L. cremoris* NZ9000, and their effect on cell growth and the release of cytoplasmic proteins into the extracellular environment was investigated. The prophage proteins were either tagless or fused with the tags Myc (holin) or 8×His (endolysin), either at the N- or the C-terminus, to allow their detection (Figs. 1a and 2a).

Initially, we examined how the tags affected prophage protein production and activity. When holin gene expression was induced at the beginning of cell growth, a considerable growth inhibition (lasting 7 h) was observed in the strains producing the tagless holin and holin with the C-terminal Myc tag (Fig. 1b). The growth of the strain producing the holin with N-terminal Myc was less inhibited and similar to the growth observed for the control strain transformed with an empty plasmid pNBBX in the presence of nisin. This slight growth inhibition was likely due to the toxicity of the inducer, bacteriocin nisin. When holin production was induced in the middle of the exponential growth phase, the pronounced growth inhibition was still observed for the strain expressing the C-terminally tagged holin (Fig. S3a), while the other strains were less inhibited compared to when induced at the beginning of growth. Reduced growth rate for the C-terminally tagged holin induced in the mid-exponential growth phase was less pronounced when the experiment was conducted in tubes instead of the microplates (Fig. S6a), although lower final optical densities were observed compared to the control strain. For further experiments, we therefore decided to induce holin production in the middle of the exponential growth phase. Western blot analysis of the induced cell lysates indicated only the C-terminally tagged holin was produced, while the N-terminally tagged holin variant was not detected (Fig. 1c). In line with this result, only the tagless holin and the C-terminally tagged holin were active, as evident by the increased amount of cytoplasmic proteins released to the extracellular medium relative to the control strain (Fig. 1d). These results are also consistent with the growth curves showing the pronounced inhibitory effect of the C-terminally tagged holin variant as described above (Fig. 1b, S3a). Altogether, the results indicate that N-terminal tagging of holin impairs its production or stability. No holin was detected in the growth medium (Fig S1), suggesting that the presence of intracellular proteins in the medium (Fig. 1d) are due to the increased membrane permeability and not extended cell lysis. Since the C-terminally tagged holin was successfully detected with WB, and its activity was comparable or even higher than that of holin without the tag, we decided to use the C-terminally tagged holin for the design of all further holin constructs.

In order for prophage endolysin to reach its site of action, it has to be translocated through the cell membrane into the cell wall. According to the SignalP − 6.0 tool, tested prophage endolysin possesses no signal sequence for secretion, making it entirely dependent on transport through the pores created by its partner protein holin. Therefore, two versions of constructs for endolysin expression were designed; one with the addition of the secretion signal peptide of the endogenous Usp45 protein (SP_Usp45_) and the other without it (Fig. 2a). When the endolysin production was induced at the beginning of growth, all strains with endolysin showed a growth inhibition lasting around 8 to 9 h. The control strain also exhibited slower growth upon the addition of the inducer bacteriocin nisin, which can be attributed to its toxic effect, but the effect was less pronounced (Fig. 2b). When expression of endolysin gene was induced in the middle of the exponential growth phase, no growth inhibition was observed in any of the constructs (Fig. S3). In further experiments, the expression of endolysin gene was therefore induced in the middle of the exponential growth phase. Western blot analysis of the cell lysates of overnight cultures revealed the presence of all variants of endolysin (containing either N- or C-terminal His8 tag). However, when the intensities of endolysin bands in Western blot were compared to those in SDS-PAGE, it was evident that endolysin variants with a C-terminal tag were detected more efficiently with antibodies than those with an N-terminal tag (Fig. 2c). Western blot of the conditioned medium (Fig. 2d, S2) also demonstrated that the position of his-tag influences the secretion efficiency of endolysin. Only the endolysin variants containing the His8 tag at its C-terminus were detected in the Western blot in concentrated conditioned medium by anti his-tag antibodies. The addition of a secretion signal peptide did not improve the endolysin secretion; on the contrary, less endolysin was detected on the cell wall with the secretion signal peptide than without it (Fig. 2d). As the endolysin variant lacking the secretion signal peptide and containing the His8 tag at the C-terminus was successfully secreted from the cells and detected on the cell wall to a greater extent, we decided to use this variant for the design of all further constructs containing endolysin.

### Co-expression of genes encoding prophage lytic proteins and cellulase in *L. cremoris*

**Fig. 3:**
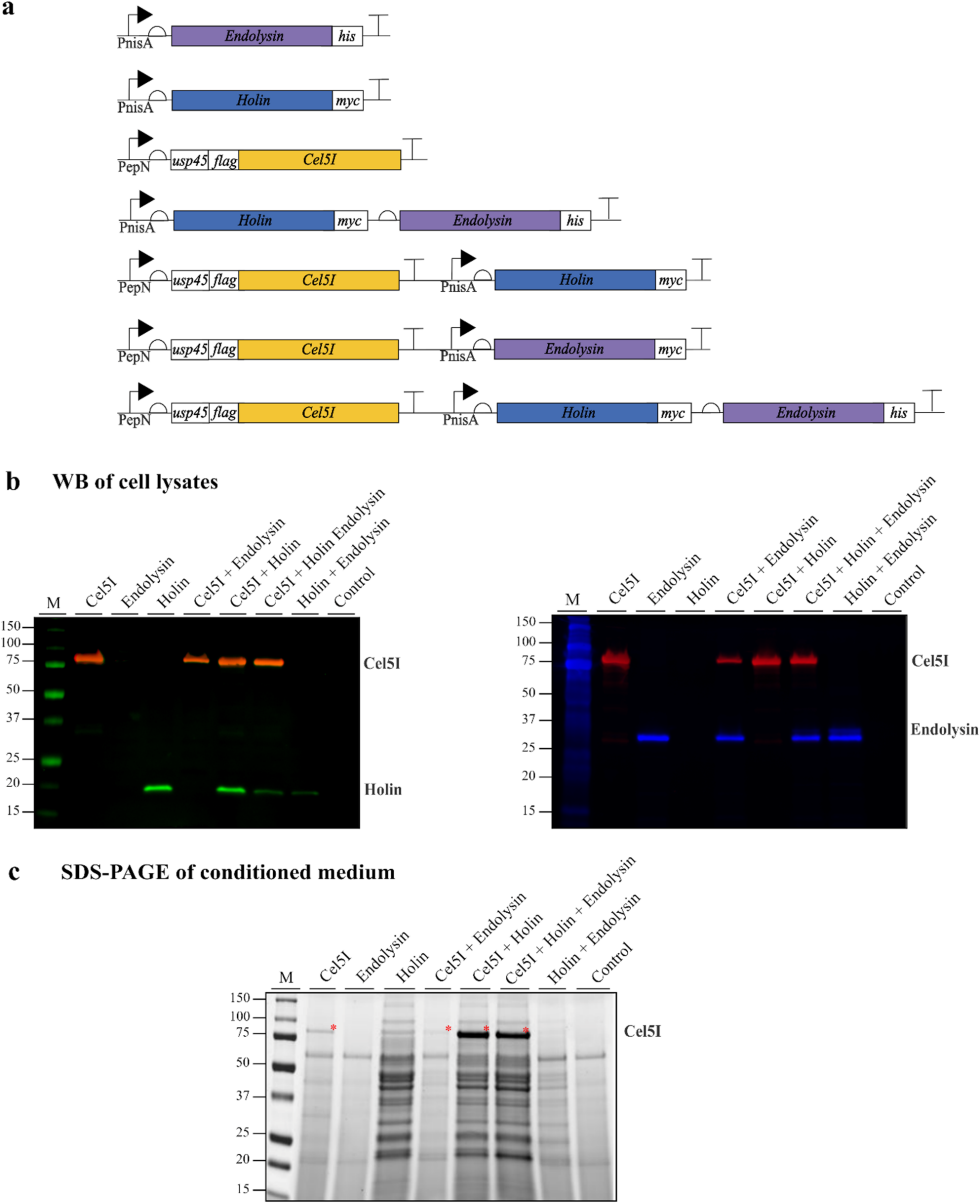
Simultaneous expression of cellulases and prophage proteins in *L. cremoris*. (**a**) Constructs were designed for inducible expression of endolysin or holin gene, constitutive expression of Cel5I cellulase gene, or co-expression of two, or of all three genes in *L. cremoris*. PnisA: nisin-inducible promoter; PepN: constitutive promoter; usp45: signal peptide sequence; his, myc and flag are protein tags. (**b**) Western blot of cell lysates and (**c**) SDS-PAGE of concentrated conditioned media from *L. cremoris* cells transformed with plasmids containing the prepared constructs. Expression of endolysin, holin or both genes was induced with 25 ng/mL nisin in the mid-exponential growth phase. Control: *L. cremoris* strain transformed with empty pNBBX plasmid. M: Molecular weight standard. Red bands on the WB correspond to the Cel5I cellulase detected with the primary rabbit anti-Flag antibody and the secondary goat anti-rabbit antibody conjugated with the fluorophore StarBright 520. Green bands on the left WB correspond to holin and blue bands on the right WB to endolysin. Holin was detected with a primary mouse anti-Myc antibody and endolysin with a primary mouse anti-his antibody. Subsequently, both were detected with the same secondary goat anti-mouse antibody conjugated to the fluorophore StarBright 700. The red asterisks on the SDS-PAGE indicate cellulase Cel5I. Endolysin denotes Endolysin_his; Holin denotes Holin_myc; Cel5I denotes flag_Cel5I

Based on the results described above, we designed further genetic constructs to investigate the effect of holin and endolysin on secretion of cellulase Cel5I (NCBI accession numbers: WP_012634873.1; 78.8 kDa) [7] in *L. cremoris*. The constructs enabled the co-expression of cellulase Cel5I gene with either holin or endolysin gene, or with both prophage genes simultaneously (Fig. 3a). Holin and endolysin contained their corresponding protein tags (Myc for holin and His8 for endolysin) at the C-terminus, whereas cellulase Cel5I possessed an N-terminal Flag tag. The cellulase Cel5I gene was under the PepN promoter to allow its constitutive expression, whereas the expression of holin and endolysin was induced with nisin in the mid-exponential growth phase. Western blot analyses of *L. cremoris* cell lysates confirmed that the expression of holin, endolysin and cellulase Cel5I was successful after co-expression of the prophage and cellulase genes from all prepared constructs (Fig. 3b, Fig. S4). The co-expression of Cel5I cellulase with holin significantly improved secretion of Cel5I compared to the expression of Cel5I alone, as shown on the SDS-PAGE gel of the concentrated conditioned medium (Fig. 3c). In contrast, co-expression of endolysin and cellulase Cel5I genes did not improve the secretion of cellulase Cel5I at all, even when the endolysin gene was expressed together with its partner protein holin (Fig. 3c). In the latter case, the genes for both prophage proteins were co-expressed under the control of a single promoter, resulting in lower expression of holin compared to when the holin gene was expressed alone or together with the Cel5I cellulase (Fig. 3b).

### Optimization of inducer concentration and induction timing for holin expression

**Fig. 4:**
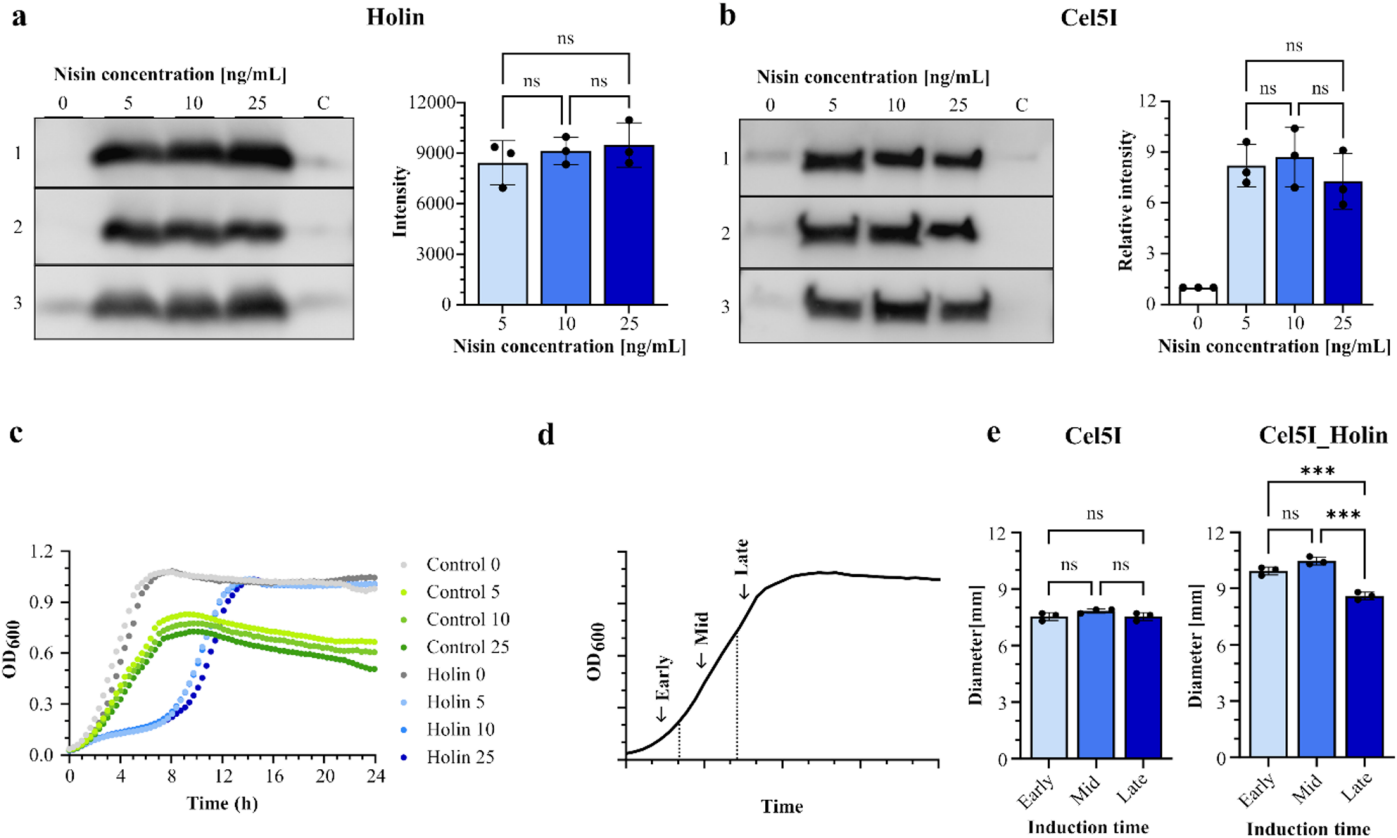
Optimization of inducing conditions for expression of holin gene in *L. cremoris*. Quantification of holin in cell lysates (**a**) and Cel5I cellulase in conditioned media (**b**) of overnight cultures, using Western blot and densitometric quantification of holin and Cel5I bands. Holin gene expression was induced with different nisin concentrations (0, 5, 10, 25 ng/mL) in the mid-exponential growth phase. For holin, raw band intensities are shown, while for Cel5I, the band intensities at different nisin concentrations were normalized to that at 0 ng/mL nisin. (**c**) Growth curves of *L. cremoris* strains transformed with an empty pNBBX plasmid (control) or a plasmid containing the holin gene with C-terminal Myc tag. Holin expression was induced with different concentrations of nisin (0, 5, 10, 25 ng/mL) at the time of inoculation. Data are presented as mean of at least three biological replicates. (**d**) The induction times of holin gene expression in the early, mid and late exponential growth phase. (**e**) Diameters of decolourisation zones on carboxymethyl cellulose agar plates stained with Congo red indicating cellulase activity in the conditioned medium of *L. cremoris* overnight cultures with (Cel5I_Holin) or without the holin gene co-expression (Cel5I) at different induction time points (in the early, mid and late exponential growth phase) with 25 ng/mL nisin. Data for (**a**), (**b**) and (**e**) are presented as mean ± standard deviation (SD) of three biological replicates. Statistical analysis was carried out using one-way ANOVA. *** *P* < 0.001; ns: not significant

We tested three different concentrations of nisin (5 ng/mL, 10 ng/mL and 25 ng/mL) for the induction of holin gene expression. All nisin concentrations enabled comparable production of holin (Fig. 4a) and had the same effect on the secretion of cellulase Cel5I in *L. cremoris* (Fig. 4b). Also, no significant differences in *L. cremoris* growth were observed when holin production was induced with different nisin concentrations (Fig. 4c). Therefore, the highest nisin concentration (25 ng/mL) was used for further experiments. Moreover, we tested the effect of three different induction time points on holin gene expression: in the early, mid and late exponential growth phase (Fig. 4d). For each induction time, the amount of secreted cellulase Cel5I present in the medium was evaluated through indirect analysis of its activity on carboxymethyl cellulose agar plate. The activity was determined by measuring the diameter of the decolourisation zone that appeared after staining with Congo red dye. A larger decolourisation zone indicates a higher amount of cellulase in the medium. There was no significant difference in the amount of secreted cellulase when holin production was induced in the early and mid-exponential growth phases (Fig. 4e, S5). However, when induced in the late growth phase, the secretion of cellulase Cel5I was significantly lower, but still higher than when the holin gene was not co-expressed. Therefore, for further experiments, the induction of holin was performed in the mid-exponential growth phase.

### Co-production of holin with cellulases to achieve their improved secretion and surface display

**Fig. 5:**
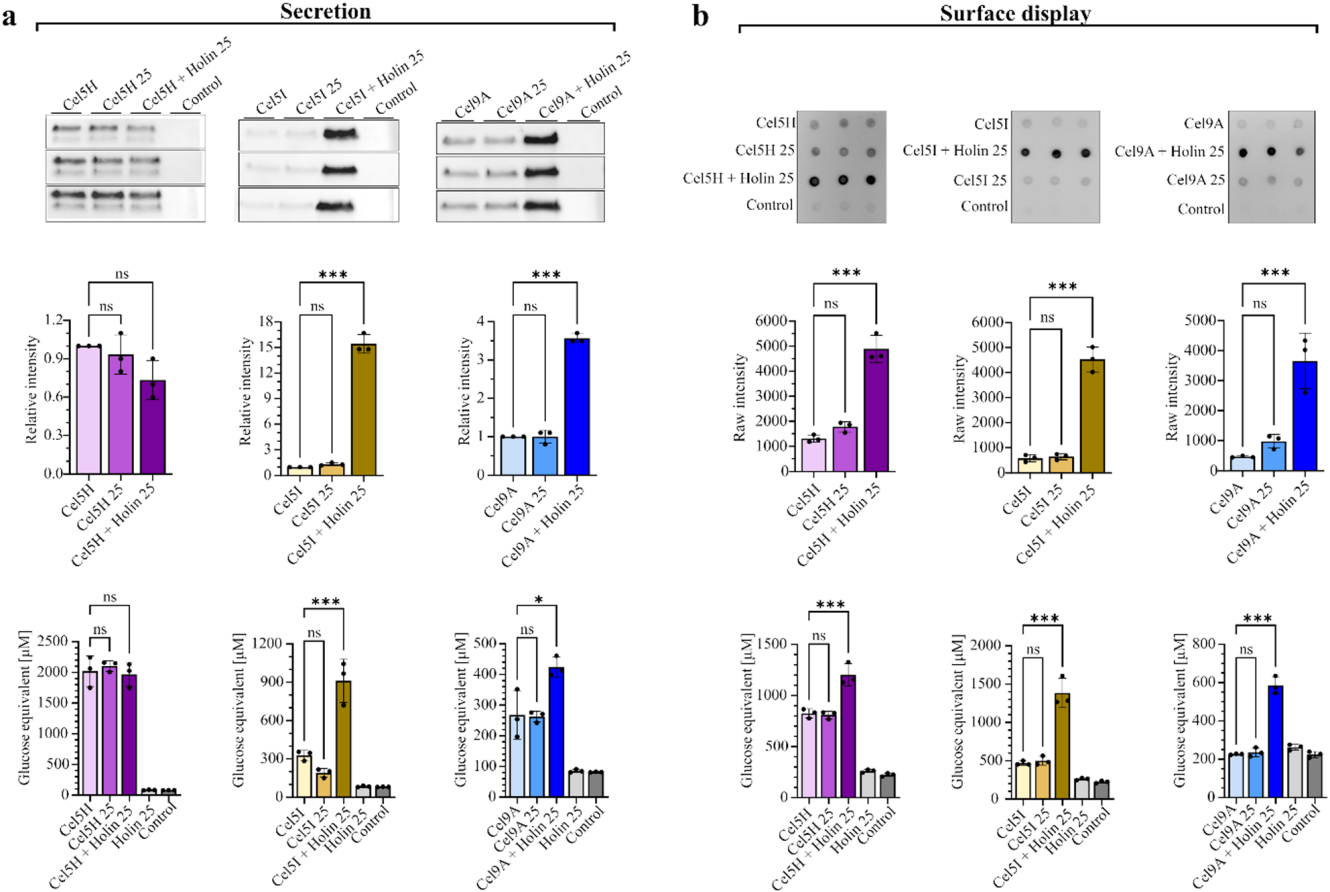
Effect of holin on cellulase secretion and surface display. (**a**) Conditioned media of *L. cremoris* overnight cultures were used for detection of cellulases (Cel5H, Cel5I and Cel9A) with Western blot (WB; upper row, with densitometric analysis in the middle row) and for assessment of their cellulase activity (lower row). The cellulase WB band intensities were normalized to the cellulase WB band intensity from the conditioned medium of *L. cremoris* cells expressing only the cellulase without the addition of nisin. For activity assays, culture media after overnight cultivation were dialysed and incubated with amorphous cellulose. (**b**) Detection of cellulases (Cel5H, Cel5I and Cel9A) displayed on *L. cremoris* cell surface with dot-blot analysis (upper row), the corresponding raw intensities of cellulases on dot-blots (middle row) and cellulase activity assays (lower row). Cellulases contained non-covalent anchor AM12 for surface display. In dot-blots, cellulases on cell surface were detected with anti-flag antibody. In activity assays, washed cells from overnight cultures were incubated with amorphous cellulose. For activity assay with surface displayed cellulases *L. cremoris* NZ3900 strain was used. The cells expressed only the cellulase (with or without nisin) or co-expressed the cellulases with nisin-induced holin. 25: addition of 25 ng/ml nisin in mid-exponential growth phase. Control: cells transformed with empty pNBBX plasmid. Activity of cells expressing holin without cellulases is also shown. Data are expressed as mean ± SD of three biological replicates. Statistical analysis was carried out using one-way ANOVA. *** *P* < 0.001; * *P* < 0.01; ns: not significant

To assess whether the developed system for enhanced protein secretion can be applied to different cellulases, genetic constructs for the co-expression of the holin gene and genes encoding cellulases Cel5H [29] and Cel9A [30] (NCBI accession numbers: WP_011469710.1; 63.3 kDa and WP_312101035.1; 104.8 kDa, respectively) were prepared. The secretion of each cellulase by *L. cremoris* was tested with or without the holin gene induction, and assessed by quantifying the cellulases in the conditioned media of the overnight cultures by Western blot. A 15-fold improvement in secretion was observed for Cel5I cellulase and a 3.5-fold improvement for Cel9A cellulase when the holin gene was co-expressed. In contrast, holin gene co-expression did not enhance the quantity of Cel5H cellulase present in the growth medium (Fig. 5a). To confirm the functionality of secreted cellulases, their activity in the conditioned media after overnight cultivation was also tested and confirmed using amorphous cellulose as a substrate. Accordingly, the overnight culture medium containing a higher amount of secreted cellulase showed a significantly higher activity than the medium with a lower amount of the same cellulase (Fig. 5a). Co-expression of the holin gene and the genes encoding cellulases fused to the non-covalent anchor AM12 was also performed to determine whether holin co-expression could also enhance the surface display of cellulases in *L. cremoris*. Using dot-blot analysis, we confirmed that the surface display of all three cellulases (Cel5H, Cel5I and Cel9A) was improved when the holin gene was co-expressed. This was further confirmed by an activity assay on amorphous cellulose that tested cells with surface displayed cellulases (Fig. 5b). Since nisin, the inducer of holin gene expression, influences membrane permeability, its independent effect on cellulase secretion and surface display of cellulases was also assessed at the levels of protein quantity and activity. However, nisin alone at the concentration we use for induction of holin production had no additional impact on the secretion and surface display of cellulases (Fig. 5a and b). To elucidate why holin enhanced Cel5H surface display (Fig. 5b) but had no apparent effect on Cel5H secretion (Fig. 5a), dot-blot analyses of intact *L. cremoris* cells expressing individual cellulases Cel5H, Cel9A and Cel5I without the AM12 anchor were performed (Fig. S7). This revealed an increased amount of Cel5H on cell surface when co-expressed with holin in comparison to other two cellulases Cel9A and Cel5I, indicating that holin facilitates Cel5H translocation across the cell membrane; however, Cel5H remains bound to the cell surface (even without the anchor). Holin therefore improves secretion also for Cel5H cellulase. Nevertheless, Cel5H amount in the growth medium is not increased with holin co-expression as observed for Cel9A and Cel5I, as secreted Cel5H attaches nonspecifically to the surface *L. cremoris* cells. This observation was further supported by Western blot analysis of lysates of whole cells expressing different cellulases with or without holin (Fig. S8b). With holin co-expression, the amount of Cel5H in lysates was significantly increased (in line with increased Cel5H on cell surface), while the amounts of Cel5I and Cel9A decreased with holin co-expression (in line with their secretion into the growth medium).

### Genome integration of holin gene and its co-expression with cellulases

**Fig. 6:**
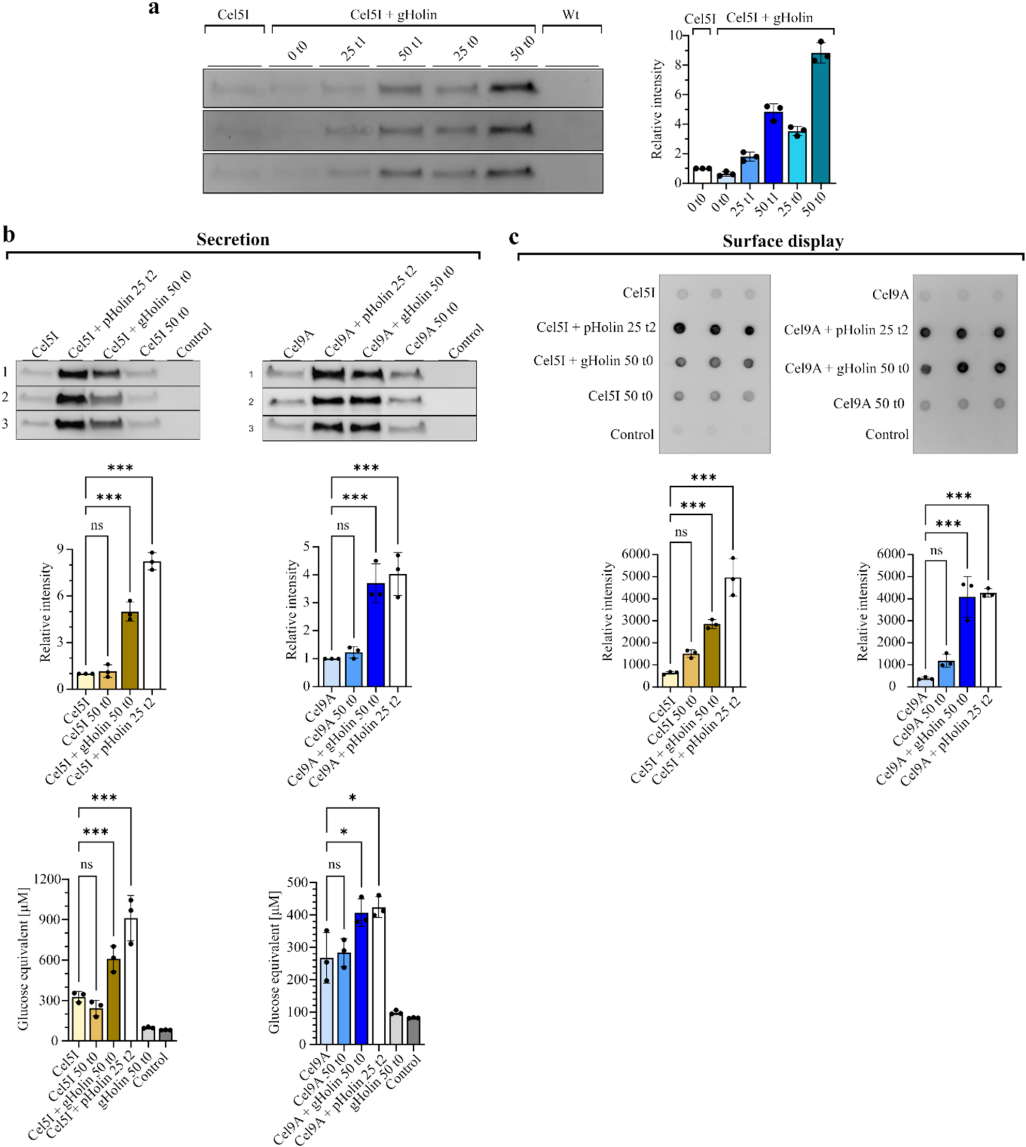
Enhancement of cellulase secretion and surface-display with genome-integrated holin. (**a**) Western blot (WB) of secreted Cel5I in the medium of overnight *L. cremoris* cultures. Cel5I was expressed either in wild-type *L. cremoris* NZ9000 strain (Wt) or in the *L. cremoris* strain with holin gene integrated into its genome (gHolin). Holin was induced with different nisin concentrations (0, 25 or 50 ng/mL) at the time of inoculation (t0) or at the early exponential growth phase (t1). The graph shows Cel5I band intensities, normalized to the intensity of Cel5I from the Wt *L. cremoris* strain. (**b**) WB of secreted Cel5I and Cel9A in the media of overnight *L. cremoris* cultures. Cellulases were expressed either alone (with or without nisin) or co-expressed with nisin-inducible holin encoded either on plasmid (pHolin) or integrated into the genome (gHolin). The graphs below WBs show intensities of cellulase bands on WB (upper row) and cellulolytic activities of dialysed conditioned media on amorphous cellulose (lower row). The cellulase band intensities were normalized to that of the corresponding cellulase secreted from *L. cremoris* in the absence of nisin and holin. (**c**) Dot-blot analyses of intact *L. cremoris* cells displaying Cel5I or Cel9A on their cell surface, detected with anti-flag antibody. The graphs below display the corresponding raw intensities of cellulases. In panels (**b**) and (**c**), gHolin was induced with 50 ng/mL nisin at the time of inoculation (t0), while pHolin was induced with 25 ng/mL nisin in the mid-exponential phase (t2). The strains containing only the cellulase (Cel5I or Cel9A) gene (without holin) were cultivated either without nisin or with 50 ng/mL nisin, added at t0. Control denotes *L. cremoris* strain transformed with empty pNBBX plasmid. Data are expressed as mean ± SD of three biological replicates. Statistical analysis was carried out using one-way ANOVA. *** *P* < 0.001; * *P* < 0.05: ns: not significant

To make the system more practical for use, the holin gene with the inducible nisin promoter was integrated into the genome of *L. cremoris* at the tRNA-Ser locus (LLNZ_t13275) using pMET306 [25]. Integration was confirmed by PCR amplification of the inserted genome region and sequencing of the amplicon. We assessed the optimal induction time and inducer concentration for holin gene expression from the genome, to achieve highest cellulase secretion. According to the Western blot analysis, the induction with 50 ng/mL nisin at inoculation was optimal for Cel5I secretion; therefore, we used this condition for further experiments (Fig. 6a). The effects of the expression of the genome-integrated holin gene on secretion (Fig. 6b) and surface display (Fig. 6c) of cellulases Cel5I and Cel9A was evaluated and compared to those of the plasmid-encoded holin. The amount of secreted cellulases in the conditioned media of overnight cultures was determined with Western blot analysis, while the amount of surface-displayed cellulases was determined with dot blot analysis of intact *L. cremoris* cells. The co-expression of the holin gene, either from the genome or from the plasmid, led to similar improvement of secretion and surface display of the active Cel9A cellulase. Co-expression of genome-integrated holin also significantly enhanced the secretion and surface display of Cel5I cellulase, resulting in the levels that were slightly lower but still comparable to those achieved with plasmid-based expression. The analyses of activity of the secreted cellulases led to the same conclusions, as both the genome-integrated and the plasmid-encoded holin led to increased cellulase activity.

### Impact of holin on cell viability

**Fig. 7:**
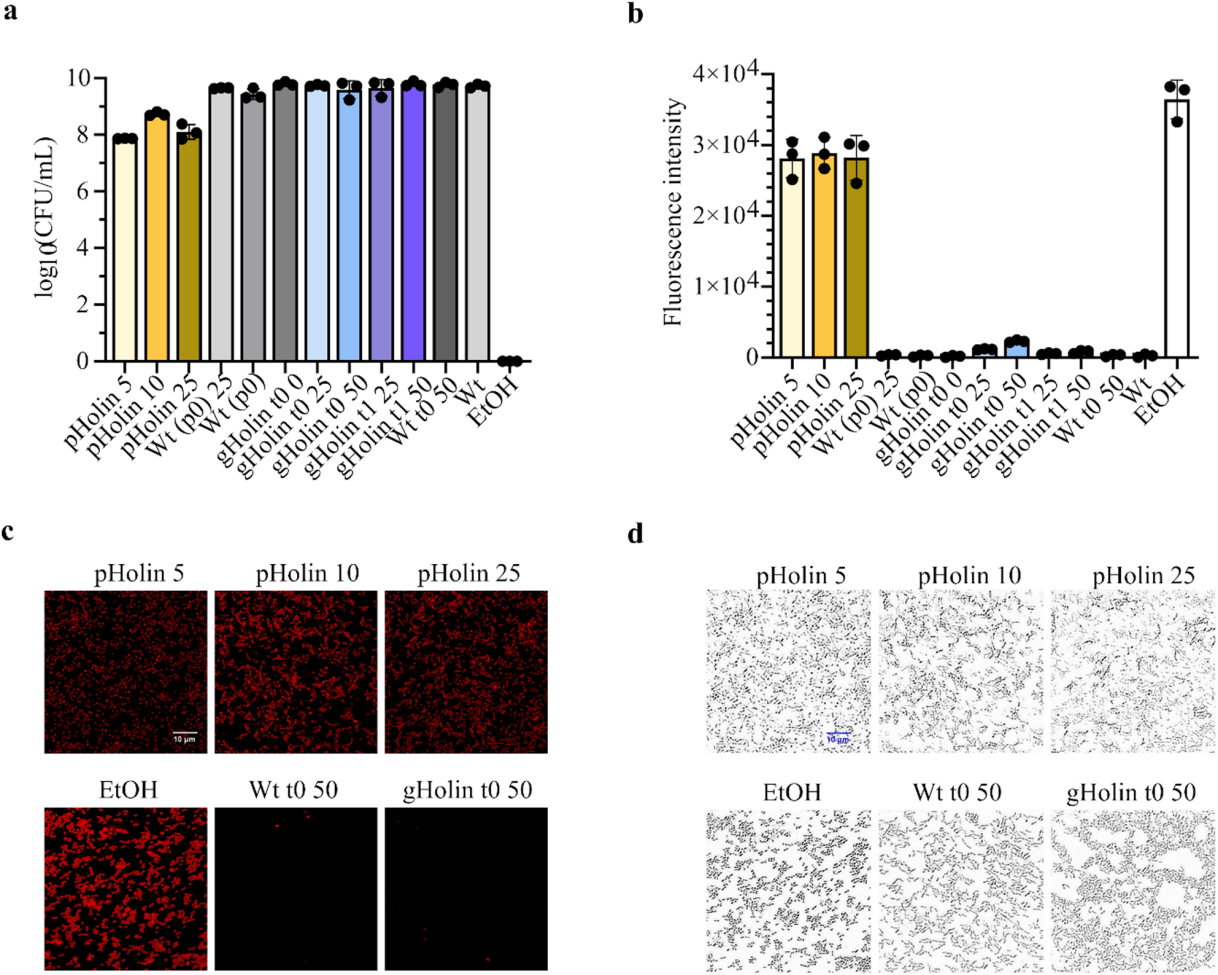
Viability of *L. cremoris* strains expressing the holin gene either from plasmid or genome. (**a**) Colony forming units per mL (CFU/mL) in cultures expressing holin under different conditions. (**b**) Spectrophotometric measurements of fluorescence intensity and representative confocal microscopy images ((**c**) fluorescence and (**d**) bright field) of propidium iodide-stained bacterial cells expressing holin under different conditions. pHolin: *L. cremoris* cells expressing the holin gene from a plasmid induced with different concentrations of nisin (5, 10, 25 ng/mL) in the mid-exponential growth phase. Wt (p0): *L. cremoris* NZ9000 cells transformed with the empty plasmid pNBBX, with or without the addition of 25 ng/mL nisin in the mid-exponential growth phase. gHolin: *L. cremoris* cells expressing the holin gene from the genome, induced with different concentrations of nisin (25 or 50 ng/mL) at different time points (t0: at inoculation or at t1: early exponential growth phase). Wt: *L cremoris* NZ9000 strain with or without the addition of 50 ng/mL nisin at the time of inoculation (t0). EtOH: *L cremoris* wt cells treated with 70% ethanol. Values in (**a**) and (**b**) are expressed as mean ± SD of three biological replicates

As observed, recombinant holin makes the cell membrane non-specifically permeable for several cytoplasmic proteins (Fig. 1d) and, presumably, also for some other intracellular molecules that could be essential for cell viability. This led us to evaluate the impact of holin gene expression, either from the plasmid or from the genome, on cell viability. This was evaluated by determining the number of colony forming units (CFU) per mL in overnight cultures expressing holin under various inducing conditions (time of induction and inducer concentration) and by staining overnight cultures with propidium iodide (PI). Cells treated with 70% ethanol were used as a positive control for viability decrease. For cells that expressed the genome-integrated holin, no significant reduction in viability was observed, while the cells expressing plasmid-encoded holin showed a decrease in viability of 1 to 1.5 log CFU/mL under the conditions used (Fig. 7a). Viability was compared with the permeability of the cell membrane for PI. Consistent with the viability results, the cells treated with 70% ethanol (no viability) exhibited the highest permeability for PI. Likewise, the results for overall membrane permeability of cells that expressed holin from the genome and from plasmid were (Fig. 7b), in line with the viability results, with cells expressing holin gene from the plasmid showing higher permeability compared to cells with genome-integrated holin gene. Microscopy images were consistent with spectroscopic fluorescence measurements of propidium iodide-stained cells with altered membrane permeability (Fig. 7b, c). Furthermore, decline in cell viability following holin induction is reversible, as removal of nisin (the inducer of holin gene expression) from the growth medium after 20 h of cultivation leads to increase in viable cell counts to the level equal to that of cultures grown in the absence of nisin (Fig. S8a).

## Discussion

Genetically engineered *L. cremoris* NZ9000 strains producing heterologous cellulases Cel5I, Cel5H and Cel9A that originate from cellulolytic bacteria *Ruminiclostridium cellulolyticum*, *Saccharophagus degradans* and *Lachnoclostridium phytofermentans*, respectively, had previously been identified as promising candidates for developing bioprocesses to convert cellulosic plant biowaste into high-value organic compounds [12]. To further enhance the cellulolytic capability of the engineered *L. cremoris* strains, this study aimed to improve the cellulase secretion capacity of *L. cremoris*. To achieve this, we have tested several approaches that had previously been successfully applied for the improvement of recombinant protein secretion in *L. cremoris* [15–17, 19]. Among them were the use of *L. cremoris* strain with inactivated endogenous extracellular HtrA protease (Fig. S9b, S9d) and fusion of different short peptide sequences with negative net charge between the signal peptide and the mature protein (Fig. S9a, S9c). However, none of these approaches led to a significant improvement in cellulase secretion. This suggests that degradation of cellulases by extracellular HtrA protease and insufficient recognition/ processing of signal peptide by the Sec transport system are probably not the limiting factors in cellulase secretion in *L. cremoris*. Another bottleneck in recombinant protein secretion might be saturation of the Sec transport machinery by overproduced proteins. There have been only a few reports of increasing the unspecific permeability of *L. cremoris* cell wall and cell membrane to bypass the limitations of the endogenous protein secretion pathway [20, 31], but they indicated notable improvement. This study therefore attempted to make the *L. cremoris* cells permeable for cellulases Cel5I, Cel5H and Cel9A by overproducing the lytic prophage proteins holin and endolysin encoded in *L. cremoris* NZ9000 genome, a strategy similar to that used by Guo et al. [20]. The overproduced holin itself significantly improved secretion of recombinant cellulases (Figs. 3, 5 and 6), similar to what was observed for other recombinant proteins by Guo et al. [20]. Interestingly, in study by Guo et al. [20], the overproduction of holin did not lead to leakage of intracellular proteins into extracellular environment, which is in contrast to our findings (Fig. 1d). Our results suggest that the presence and position of a protein tag on holin contributes to its amount produced and the resulting activity (Fig. 1). Presumably, the type of protein tag used by Guo et al. [20] (S-tag) could have affected holin oligomerization and pore formation in a manner different than the Myc tag used in this study. Guo et al. [20] proposed two possible roles for holin in the secretion of target recombinant proteins: a direct role through pore formation, as supported by our data, and an indirect role by promoting the alternative SRP pathway.

In addition to holin, we also tested the effect of endolysin on cellulase secretion. The prophage endolysin employed in this system is, according to the Pfam database, a lysozyme with peptidoglycan N-acetylmuramoylhydrolase activity. Overproduction of this endolysin alone or in combination with its partner protein holin did not notably improve cellulase secretion (Fig. 3c) nor did it lead to cell lysis, regardless of our attempts to optimize its expression by testing different endolysin variants (with or without the His-tag at N- or C-terminus, addition of signal peptide) (Fig. 2) or induction at different exponential growth stages (Fig. S5). However, it delayed the growth of *L. cremoris* NZ9000 cultures (Fig. 2b) and bound to the cell wall of *L. cremoris* (Fig. 2d). It appears that the amount of endolysin produced in cells might be insufficient for detectable leakage of cytoplasmic proteins. To enhance the cell wall digestion, Liu et al. [32] co-produced two endolysins (lysozyme and transglycosylase) in *Synechocystis* sp. PCC 6803, digesting peptidoglycan at two different sites. However, this led to slower growth of the strain carrying these two recombinant genes. There are another two possible explanations for not observing enhanced cellulase secretion by co-expression of the prophage endolysin gene. Lesions in the cell wall produced by endolysin could be too small to release the cellulases into the extracellular environment, or the transport across the cell wall does not present a significant limitation in cellulase secretion in *L. cremoris*. This reasoning might explain why endolysin did not further increase the impact of holin on cellulase secretion.

Another strategy in developing cellulolytic *L. cremoris* cells is surface display of cellulases. This means the cellulases produced move across the cell membrane and attach to the cell wall on the outer cell surface via an anchor domain, instead of freely secreting into the extracellular media. Surface-displayed proteins also employ the classical protein secretion pathways such as Sec [33], suggesting that secretion might also present one of the limiting factors in achieving an efficient surface display [34, 35]. To our knowledge, there have been no reports on using holin as a tool to enhance the surface display of recombinant proteins on bacterial cell wall. Our results show that co-production of holin with cellulases containing a cell wall anchoring domain lead to higher amount of cellulases displayed on the cell surface. Presumably, holin alters the permeability of cell membrane, while the cell wall likely stays intact, enabling proteins with a cell wall binding domain to bind to the cell surface. We thus show that holin might also be employed for improving the surface-display of target proteins.

Interestingly, while holin markedly improved secretion of cellulases Cel5I and Cel9A, the amount of cellulase Cel5H in the growth medium was not increased when co-expressed with holin. This was due to the propensity of Cel5H to attach onto *L. cremoris* cell surface when secreted with the aid of holin (Fig. S7). This affinity of Cel5H towards cell surface even in the absence of anchoring domain was also observed in previous study [12]. Presumably, Cel5H might be unstable or partially misfolded when secreted, leading to its non-specific interaction with cell surface. However, the exact reasons for this interaction are left to be examined.

For co-expression of the holin gene with cellulase genes, we employed the BglBrick approach [22] and assemble the expression cassettes using the pNBBX plasmids. This approach allows an easy assembly of multiple expression cassettes, making it convenient also for other recombinant proteins whose secretion capacity is limited. In some cases, the stable cloning and co-expression of multiple plasmid-encoded proteins is difficult to achieve. Therefore, we also prepared *L. cremoris* NZ9000 strain with the gene encoding holin integrated into its genome under a strong inducible promoter to allow its controlled expression (*L. cremoris* NZ9000-Holin). This strain enabled improved cellulase secretion (Fig. 6b), without any major loss in cell viability, as opposed to the strain with plasmid-encoded holin (Fig. 7). Since expression of holin integrated in the genome was induced at the beginning of the growth, the growth of these strains was slower compared to the strains that expressed holin from the plasmid, induced in the mid-exponential growth phase. However, the strains expressing holin either from the genome or from the plasmid reached comparable optical densities at the end of the growth period (Fig. S6). While secretion of Cel9A was comparable regardless of whether holin was expressed from the genome or the plasmid, the level of secreted Cel5I achieved with genome-integrated holin was slightly lower than that achieved with plasmid-encoded holin (Fig. 6b). It is likely that more holin is expressed from the high-copy pNBBX plasmid than from a single holin gene in the genome. Higher amounts of holin expressed from plasmid appear to improve the secretion of Cel5I, whereas the secretion efficiency of Cel9A plateaued already with genomic holin expression. Altogether, this makes the strain with genome-integrated holin preferable in applications where cell viability needs to be maintained, while the plasmid-encoded holin might be preferred in applications that aim for maximal protein secretion output.

Holin co-expression can significantly enhance the nonspecific secretion of intracellular proteins by partially bypassing signal-peptide (SP)–dependent limitations through increased membrane permeability. Consequently, this strategy might be more effective than optimization of the secretion signal peptide or modification of the local charge at the signal peptide–mature protein junction aimed at improving signal peptidase recognition. Insertion of short negatively charged peptide sequences between the signal peptide and the mature protein can alter N-terminal processing or protein activity, and its effectiveness is highly protein-dependent. However, holin co-expression does not address limitations related to extracellular protein degradation, incorrect folding, or poor solubility. When these factors are the primary bottlenecks, holin expression is unlikely to increase the yield of active secreted protein. In such cases, inactivation of the extracellular housekeeping protease HtrA or co-expression of molecular chaperones is expected to be more effective. Despite its potential benefits, holin co-expression also has several drawbacks. The increased membrane permeability might lead to co-release of intracellular proteins, resulting in contamination of the target product and complicating downstream purification if needed. In addition, holin-mediated leakage of cytosolic proteases may be detrimental for proteins that are sensitive to intracellular proteolytic degradation. Finally, holin expression can disrupt normal cellular physiology, negatively affecting cell viability and other essential cellular processes.

The present study primarily focuses on a proof-of-principle approach to enhance secretion of a target recombinant protein in *L. cremoris* through co-expression of a prophage protein under batch cultivation conditions. Considering the potential application of this strategy in scaled-up processes, the cost of the inducer may become a limiting factor. Moreover, because nisin is a bacteriocin, lower inducer concentrations are preferable. We therefore evaluated and confirmed that reduced concentrations of the holin inducer nisin (as low as 5 ng/mL) are sufficient to induce holin expression from the genome and achieve secretion levels comparable to those obtained with higher inducer concentrations (25 ng/mL) (Fig. 4). We have also shown that upon inducer removal, viability of cells is restored (Fig. S8a) while increased secretion of cellulases is retained (Fig. S8c). Nevertheless, as this aspect was addressed only to a limited extent in the present study, further optimization of holin expression will be required to enable process scale-up, continuous cultivation, and long-duration production processes.

## Conclusion

Simultaneous expression of the prophage protein holin and heterologous cellulases in *L. cremoris* significantly improves secretion of cellulases Cel5I and Cel9A and surface display of cellulases Cel5I, Cel9A and Cel5H. The developed expression system, in which the holin gene is located either on the expression plasmid or integrated in the genome could easily be employed to enhance the secretion of other heterologous cellulases or recombinant proteins in general, especially when reduced secretion is primarily due to oversaturation of the Sec transport machinery. This finding could help overcome limitations in cellulase secretion in the genetically engineered *L. cremoris* strains, and facilitate their application in consolidated production of high-value organic compounds from plant biowaste or in ensiling processes.

## Supplementary Information

Below is the link to the electronic supplementary material.

Supplementary Material 1

## Data Availability

The datasets used and/or analysed during the current study are available from the corresponding author on reasonable request.

## Abbreviations

LAB: Lactic acid bacteria
SRP: Signal-recognition particle
SDS-PAGE: Sodium dodecyl sulfate polyacrylamide gel electrophoresis
WB: Western blot
TCA: Trichloroacetic acid
DTT: Dithiothreitol
PBS: Phosphate-buffered saline
TBST: Tris-buffered saline containing 0.05% Tween-20
PASC: Phosphoric acid swollen cellulose
CMC: Carboxymethyl cellulose
CFU: Colony-forming units
