# Supplementary material for: Co-expression of holin gene improves heterologous cellulase secretion and surface display by lactic acid bacteria Lactococcus cremoris
Source: J Biol Eng. 2026 Jan 19;20:32. doi: 10.1186/s13036-026-00625-0 (PMC12903410; doi:10.1186/s13036-026-00625-0)
Supplement: Supplementary file 1 — Supplementary Material 1 [file 13036_2026_625_MOESM1_ESM.docx]

**Supplementary Material**

**Heterologous cellulase secretion and surface display by lactic acid bacteria *Lactococcus cremoris* is improved by co-expression of holin gene**

Petra Štravs^1, 2^, Lara Repar^1, 4^, Henri-Pierre Fierobe^3^, Stéphanie Perret^3^, Aleš Berlec^1, 4^

^1^ Jožef Stefan Institute, Department of Biotechnology, Ljubljana, Slovenia

^2^ Interdisciplinary Doctoral Study Program in Biosciences, University of Ljubljana, Ljubljana, Slovenia

^3^ Aix-Marseille Université, CNRS, LCB-UMR7283, Marseille, France

^4^ Faculty of Pharmacy, University of Ljubljana, Ljubljana, Slovenia

*Construction of the expression plasmids*

Amplification of genes and other DNA sequences was performed using Phusion High–Fidelity DNA Polymerase (Thermo Scientific). Amplified DNA fragments were inserted into plasmid using Fast Digest restriction enzymes (Thermo Scientific) and T4 DNA ligase (New England Biolabs). To purify digested and amplified DNA fragments, NucleoSpin Gel and PCR Clean-up kit (Macherey and Nagel) were used. To create plasmid for expression of holin and endolysin, the genes encoding holin (GenBank: ADJ61052.1) and endolysin (GenBank: ADJ61051.1) were amplified from *L. cremoris* NZ9000 genome using primers that enabled fusion of the recombinant protein with a protein tag positioned at either the N terminus or C-terminus, or absence of tag. Amplified nucleotide sequences were inserted between the restriction sites NcoI/XbaI into the plasmid pNBBX [1]. Holin was fused with protein tag Myc (amino acid sequence: EQKLISEEDL) and endolysin was fused with protein tag 8×His (amino acid sequence: HHHHHHHH). For expression of endolysin fused with N terminal secretion signal peptide of the endogenous Usp45 protein (SPU_sp45_), the amplified endolysin gene (with or without the sequence encoding the His tag at 5’ or 3’ end) was first inserted between the restriction sites BamHI/XbaI into the plasmid pPepNsp_FCel5I [2]. The obtained DNA fragment encoding endolysin (with or without the sequence encoding the His tag) fused with the sequence encoding the N-terminal SP_Usp45_ was subsequently inserted into the pNBBX plasmid via NcoI/XbaI restriction sites. To obtain plasmid for polycistronic expression of holin and endolysin with protein tag located on C-terminus, the DNA fragments Pnis_Holin_Myc (containing sequences for PnisA promotor and holin gene with Myc tag on 3’ end, amplified from the pNBBX plasmid) and Endo_His_TT (containing sequence for endolysin gene with His tag on 3’ end and transcription terminator, amplified from the pNBBX plasmid) were fused using asymmetric overlap extension PCR method as previously described by Xiao et al. [3]. Obtained DNA fragment was inserted in plasmid pNBBX between restriction sites NheI/XhoI. The pNBBX plasmids that encode cellulases Cel5I, Cel9A and Cel5H were produced by amplifying and inserting the DNA fragment containing the PepN constitutive promoter, SP_Usp45_, cellulase gene with Flag tag on the 5’ end, a covalent cell wall anchor on 3’ end (optionally) and a transcription terminator from pPepNsp_FCellulase plasmids reported by Štravs et al. [2]. BglBrick approach, described by Plavec et al. [1] was used to construct plasmids for co-expression of cellulase gene with holin gene, endolysin gene, or with both by employing pNBBX plasmids generated in this study.

Table 1: Bacterial strains, plasmids and DNA primers used in the study. All plasmids harbour the *cat* gene encoding chloramphenicol acetyltransferase for chloramphenicol resistance. *sp_Usp45_*: signal peptide sequence of the Usp45 protein, enabling protein secretion; *flag-tag*, *myc-tag*, *his-tag*: epitopes used for immunodetection.; *c-am12*: sequence encoding the surface anchor domain cAM12. Primers labeled with F are forward primers, while those labeled with R are reverse primers. Nucleotide sequences for Myc, Flag and His epitopes, present in primers for fusion with sequence of protein gene, when amplified are bolded.

| **Strain** | **Genotype** |  | **Reference** |
| --- | --- | --- | --- |
| *E. coli* Oneshot TOP10F' | *F´{lacIq Tn10 (TetR)} mcrA ∆(mrr-hsdRMS-mcrBC) Φ80lacZ∆M15 ∆lacX74 recA1 araD139 ∆(ara-leu)7697 galU galK rpsL endA1 nupG* |  | Invitrogen |
| *L. cremoris* NZ9000 | MG1363; *pepN*::*nisRK* |  | NIZO |
| *L. cremoris* NZ3900 | MG5267 derivative (strain MG1363 containing lac operon in the chromosome); ΔlacF, pepN::nisRK |  | [4, 5] |
| *L. cremoris* NZ9000-Holin | NZ9000; *tRNA-Ser*::*holin_LLH* |  | This study |
| **Plasmid** | **Features** |  | **Reference** |
| pNBBX | pNZ8148 containing NheI, BglII, BclI and XhoI restriction sites |  | [1] |
| *L. cremoris* NZ9000 Δ*htrA* | Emr, *htrA* disrupted by single-crossover recombination |  | [6] |
| pMET306 | pMC1::core-*attP_4_*; Erm^R^ |  | [7] |
| pMET306_Pnis_HolinM_TT | pMET306 containing HolinM cassette |  | This study |
| pPepNsp_FCel9A | pNZ8148 containing gene fusion of *sp_Usp45_*, *flag-tag* and *cel9A*; P_nisA_ promoter is replaced with P_pepN_ promoter |  | [2] |
| pPepNsp_FCel5H | pNZ8148 containing gene fusion of *sp_Usp45_*, *flag-tag* and *cel5H*; P_nisA_ promoter is replaced with P_pepN_ promoter |  | [2] |
| pPepNsp_FCel5I | pNZ8148 containing gene fusion of *sp_Usp45_*, *flag-tag* and *cel5I*; P_nisA_ promoter is replaced with P_pepN_ promoter |  | [2] |
| pPepNsp_FCel9A_AM12 | pNZ8148 containing gene fusion of *sp_Usp45_*, *flag-tag, Cel9A* and *c-am12*; P_nisA_ promoter is replaced with P_pepN_ promoter |  | [2] |
| pPepNsp_FCel5H_ AM12 | pNZ8148 containing gene fusion of *sp_Usp45_*, *flag-tag, Cel5H* and *c-am12*; P_nisA_ promoter is replaced with P_pepN_ promoter |  | [2] |
| pPepNsp_FCel5I_ AM12 | pNZ8148 containing gene fusion of *sp_Usp45_*, *flag-tag*, *Cel5I* and *c-am12*; P_nisA_ promoter is replaced with P_pepN_ promoter |  | [2] |
| pNBBX_Pnis_Holin | pNBBX with Holin cassette containing *holin* gene |  | This study |
| pNBBX_Pnis_MHolin | pNBBX with MHolin cassette containing gene fusion of *holin* gene with *myc-tag* on N-terminus |  | This study |
| pNBBX_Pnis_HolinM | pNBBX with HolinM cassette containing gene fusion of *holin* gene with myc-tag on C-terminus |  | This study |
| pNBBX_Pnis_Endolysin | pNBBX with Endolysin cassette containing *endolysin* gene |  | This study |
| pNBBX_Pnis_HEndolysin | pNBBX with HEndolysin cassette containing gene fusion of *endolysin* gene with *his-tag* on N-terminus |  | This study |
| pNBBX_Pnis_EndolysinH | pNBBX with EndolysinH cassette containing gene fusion of *endolysin* gene with *his-tag* on C-terminus |  | This study |
| pNBBX_Pnis_Usp45_Endolysin | pNBBX with UspEndolysin cassette containing gene fusion of *sp_Usp4_*_5_ and *endolysin* gene |  | This study |
| pNBBX_Pnis_ Usp45_HEndolysin | pNBBX with UspHEndolysin cassette containing gene fusion of *sp_Usp45_* and *endolysin* gene with *his-tag* on N-terminus |  | This study |
| pNBBX_Pnis_ Usp45_EndolysinH | pNBBX with UspEndolysinH cassette containing gene fusion of *sp_Usp45_* and *endolysin* gene with *his-tag* on C-terminus |  | This study |
| pNBBX_Pnis_HolinM_EndolysinH | pNBBX with HolinM_EndolysinH cassete containing polycistronic gene of *holin* gene with *myc-tag* on C-terminus and *endolysin* gene with *his-tag* on C-terminus |  | This study |
| pNBBX_PepN_FCel5I_Pnis_HolinM | pNBBX with FCel5I cassette and HolinM cassette |  | This study |
| pNBBX_ PepN_FCel5I_Pnis_EndolizinH | pNBBX with FCel5I cassette and EndolysinH cassette |  | This study |
| pNBBX_ PepN_FCel5I_Pnis_HolinM_EndolizinH | pNBBX with FCel5I cassette and HolinM_EndolysinH cassette |  | This study |
| pNBBX_PepN_FCel5H_Pnis_HolinM | pNBBX with FCel5H cassette and HolinM cassette |  | This study |
| pNBBX_Pnis_HolinM_PepN_FCel9A | pNBBX with HolinM cassette and FCel9A cassette |  | This study |
| pNBBX_PepN_FCel5I_AM12_Pnis_HolinM | pNBBX with FCel5I_AM12 cassette and HolinM cassette |  | This study |
| pNBBX_Pnis_HolinM_PepN_FCel9A_AM12 | pNBBX with HolinM cassette and FCel9A_AM12 cassette |  | This study |
| **Primer** | **Sequence** | **Amplification** | **Reference** |
| EndoL_F_Nco | ATAACCATGGCTAACGGAATTGACATTTCCAGCTATC | *endolysin* and *endolysin_his* | This study |
| EndoL_F_BamHI | ATAAGGATCCAACGGAATTGACATTTCCAGCTATC | *sp_Usp45__endoysin and sp_Usp45__endolysin_his* | This study |
| EndoL_His_F_Nco | ATAACCATGGCT**CATCACCATCACCATCACCATCAC**AACGGAATTGACATTTCCAGCTATC | *his_endolysin* | This study |
| EndoL_His_F_BamHI | ATAAGGATCC**CATCACCATCACCATCACCATCAC**AACGGAATTGACATTTCCAGCTATC | *sp_Usp45__his_endoysin* | This study |
| EndoL_R_Xba | AAATTCTAGATTACACATTTTTACGATATAGTTTTTCCATTTGTG | *endolysin. his_endolysin_ sp_Usp45__endoysin and sp_Usp45__his_endolysin* | This study |
| EndoL_His_R_Xba | AAATTCTAGATTA**GTGATGGTGATGGTGATGGTGATG**CACATTTTTACGATATAGTTTTTCCATTTGTG | *endoysin_his and sp_Usp45__endolysin_his* | This study |
| Holin_F_Nco | ATAACCATGGATCAAAATTTAATGACAATCTTTAGCGGC | *holin and holin_myc* | This study |
| Holin_Myc_F_Nco | ATAACCATG**GAACAAAAACTTATTTCAGAAGAGGATCTT**GATCAAAATTTAATGACAATCTTTAGCGGC | *myc_holin* | This study |
| Holin_R_Nco | AAATTCTAGATTATTCTCCTTGTTCTGTTGGTTCTG | *myc_holin and holin* | This study |
| Holin_Myc_R_Xba | AAATTCTAGATTA**AAGATCCTCTTCTGAAATAAGTTTTTGTTC**TTCTCCTTGTTCTGTTGGTTCTG | *holin_myc* | This study |
| Holin_OL | CCATGGTGAGTGCCTCCTCGTTACCCAACTTAATCGCCTTAAAGATCCTCTTCTGAAATAAG | *holin_myc_endolysin_his* | This study |
| Endolysin_OL | CTTATTTCAGAAGAGGATCTTTAAGGCGATTAAGTTGGGTAACGAGGAGGCACTCACCATGG | *holin_myc_endolysin_his* | This study |
| NB-F-PpepN | AAAAAAGCTAGCATATAGATCTCTGTAAAAGCTGTCA | BglBrick expression cassettes with cellulase gene | This study |
| BX-R-TT2 | AAAAAACTCGAGATATTGATCAAACGATTATGCCGATAACTAAAC | BglBrick expression cassettes | [1] |
| NB-F-PnisA2 | AAAAAAGCTAGCATATAGATCTAGTCTTATAACTATACTGAC | BglBrick expression cassettes with holin or endolysin gene | [1] |
| Pnis_F_SalI | ATAAGTCGACTAGTCTTATAACTATACTGACAATAGAAAC | HolinM expression cassete for pMET306 | This study |
| TT-R-Kpn | AAATGGTACCTCGAAAGCGAAATCAAACG | HolinM expression cassete for pMET306 | This study |
| Pmet306_For_G | CAGCATATTTATTCATTTGTCAACATTTTTG | aligning upstream of the tRNA-Ser locus (LLNZ_t13275) in the genome | This study |


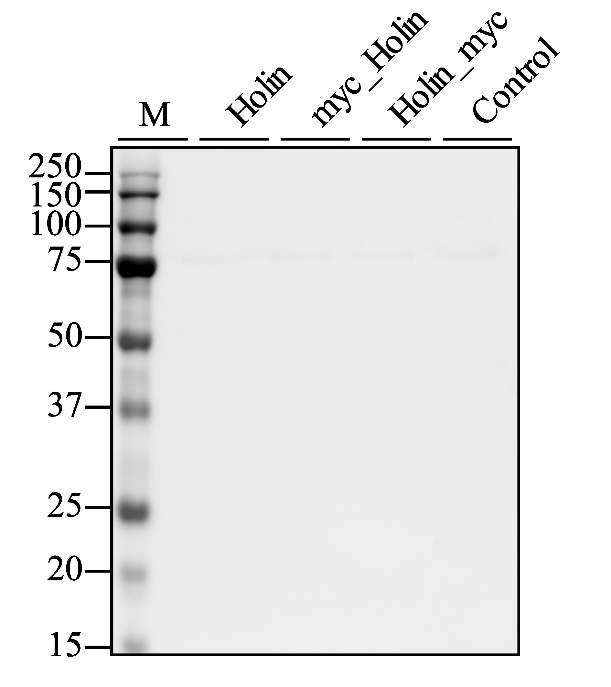


Fig. S1. Western blot of concentrated conditioned media of *L. cremoris* cells producing holin with or without the N- or C-terminal Myc-tag. Expression of holin gene was induced with 25 ng/mL nisin in the mid‑exponential growth phase. Variants without or with the N- or C-terminal Myc tag are shown as holin, myc_holin and holin_myc, respectively. Control: *L. cremoris* strain transformed with empty pNBBX plasmid. M: molecular weight standard. To detect holin, primary mouse antibodies against myc and secondary goat anti-mouse antibody conjugated to the fluorophore StarBright 700 were used.


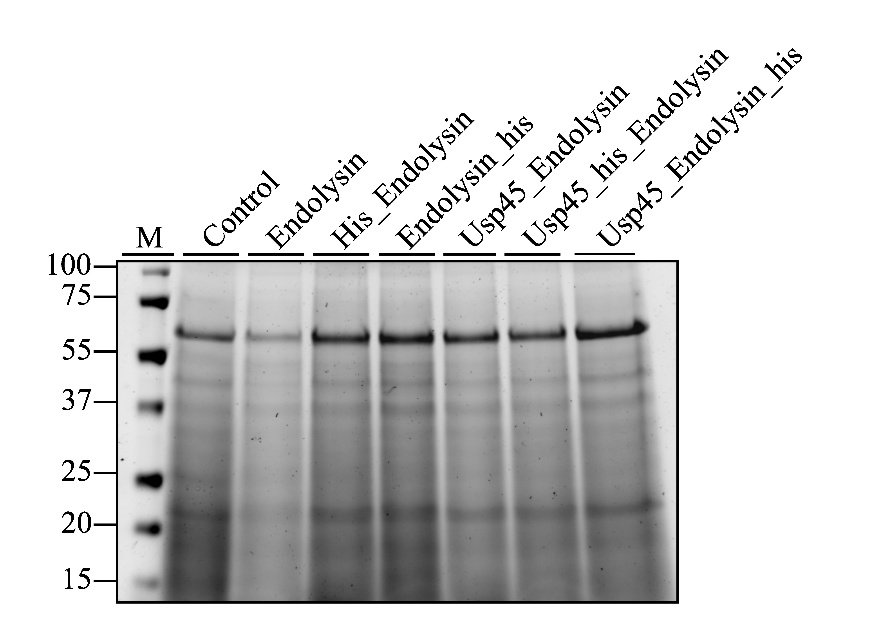


Fig. S2. SDS-PAGE gel of concentrated conditioned media *L. cremoris* cultures expressing endolysin with or without the N- or C-terminal His8 tag. Endolysin expression was induced with 25 ng/mL nisin in the mid exponential growth phase. Endolysin variants without or with the N- or C-terminal His8 tag are shown as endolysin, his_endolysin and endolysin_his, respectively. Endolysin variants with the N-terminal SP_Usp45_ signal sequence are indicated. Control: *L. cremoris* strain transformed with empty pNBBX plasmid. M: Molecular weight standard.


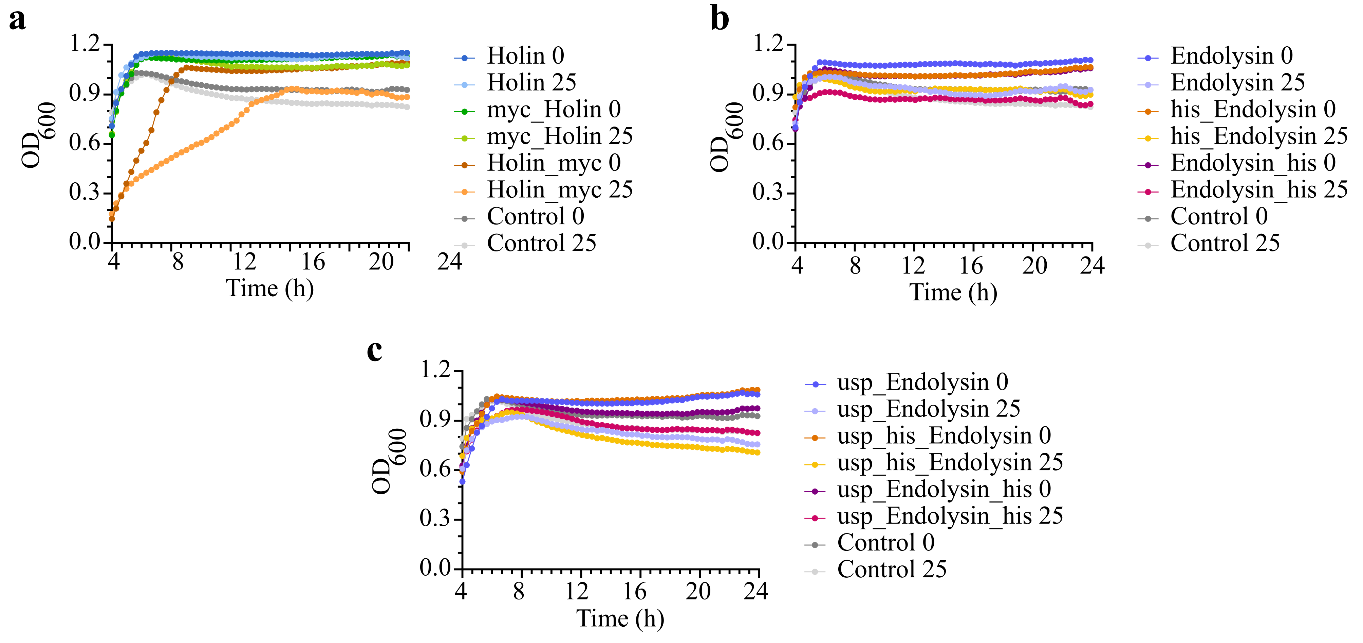


Fig. S3. Growth curves of *L. cremoris* strains expressing genes encoding prophage lytic proteins induced in the mid‑exponential phase. *L. cremoris* strains were transformed (a) with plasmids engineered for the expression of holin (untagged, or with N- or C-terminal Myc tag), (b) with plasmids engineered for the expression of endolysin, or (c) with plasmids engineered for the expression of endolysin with the secretion signal peptide sequence SP_Usp45_. Holin and endolysin expression was induced with 0 or 25 ng/mL nisin. Data are expressed as mean of three biological replicates. In all panels, control corresponds to *L. cremoris* strain transformed with empty plasmid pNBBX. This growth experiment was conducted in microplates.


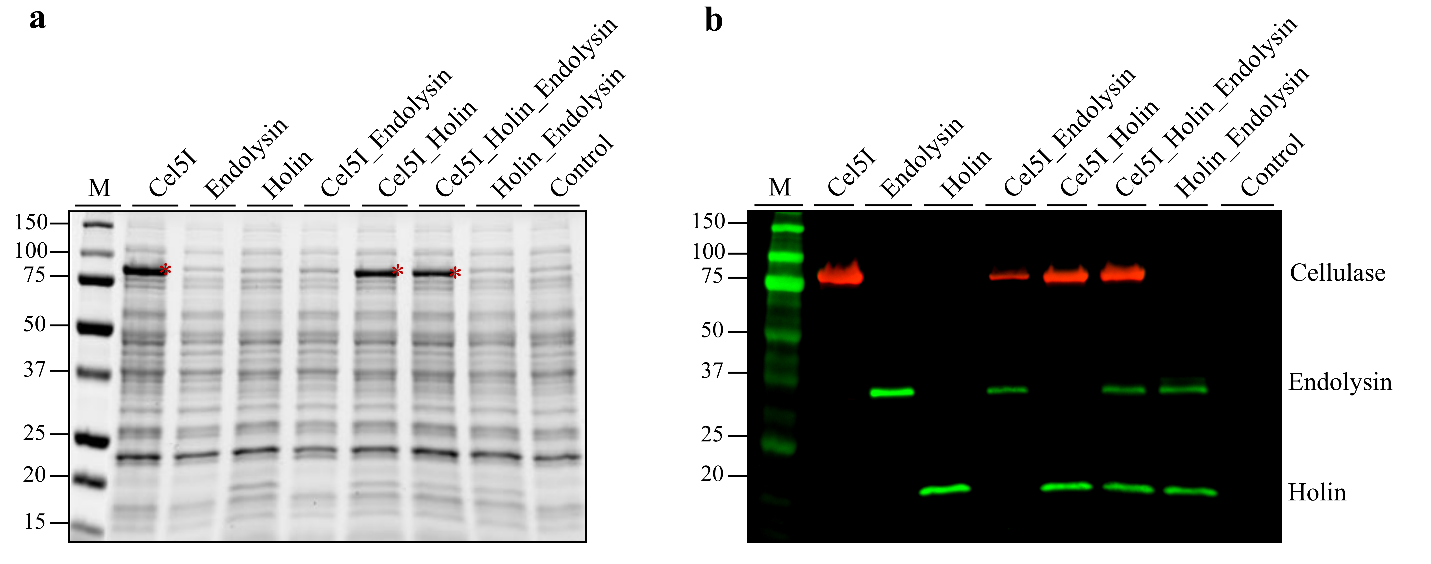


Fig. S4. Simultaneous expression of cellulase and prophage proteins in *L. cremoris*: (a) SDS-PAGE gel and (b) Western blot (WB) of cell lysates from *L. cremoris* cells expressing recombinant cellulase, endolysin or holin individually, combinations of cellulase with holin or endolysin, or all three proteins simultaneously. The cellulase gene was constitutively expressed, while the expression of endolysin, holin or both, was induced with 25 ng/mL of nisin in the mid‑exponential growth phase. Control: *L. cremoris* strain transformed with empty pNBBX plasmid. M: molecular weight standard. The red bands on the WB correspond to Cel5I cellulase detected with the primary rabbit antibody against the Flag-tag and the secondary goat anti-rabbit antibody conjugated with the fluorophore StarBright 520. The green bands on the WB correspond to holin and endolysin. Holin was detected with a primary mouse antibody against Myc-tag and endolysin with a primary mouse antibody against His-tag. Subsequently, both were detected with the same secondary goat anti-mouse antibody conjugated to the fluorophore StarBright 700. The red asterisks on the SDS-PAGE indicate cellulase Cel5I. Endolysin denotes Endolysin_his; Holin denotes Holin_myc; Cel5I denotes flag_Cel5I.
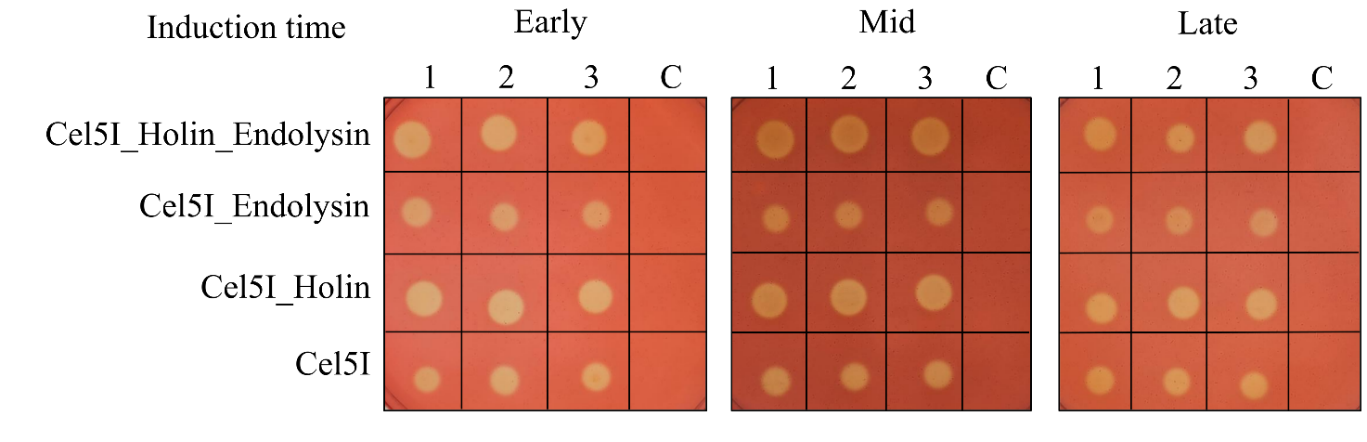
Fig. S5. Cellulase activity in conditioned media of overnight *L. cremoris* cultures, detected using carboxymethyl cellulose as substrate. The expression of the cellulase gene was constitutive, while the expression of the endolysin and holin genes was induced with 25 ng/mL nisin in the early, mid and late exponential growth phase. Biological replicates are labelled with the numbers 1, 2 and 3. Cel5I: *L. cremoris* strain expressing cellulase alone, Cel5I_Holin: *L. cremoris* strain expressing cellulase simultaneously with holin, Cel5I_Endolysin: *L. cremoris* strain expressing cellulase simultaneously with endolysin, Cel5I_Holin_Endolysin: *L. cremoris* strain expressing cellulase simultaneously with both, holin and endolysin. C: *L. cremoris* strain transformed with empty pNBBX plasmid.


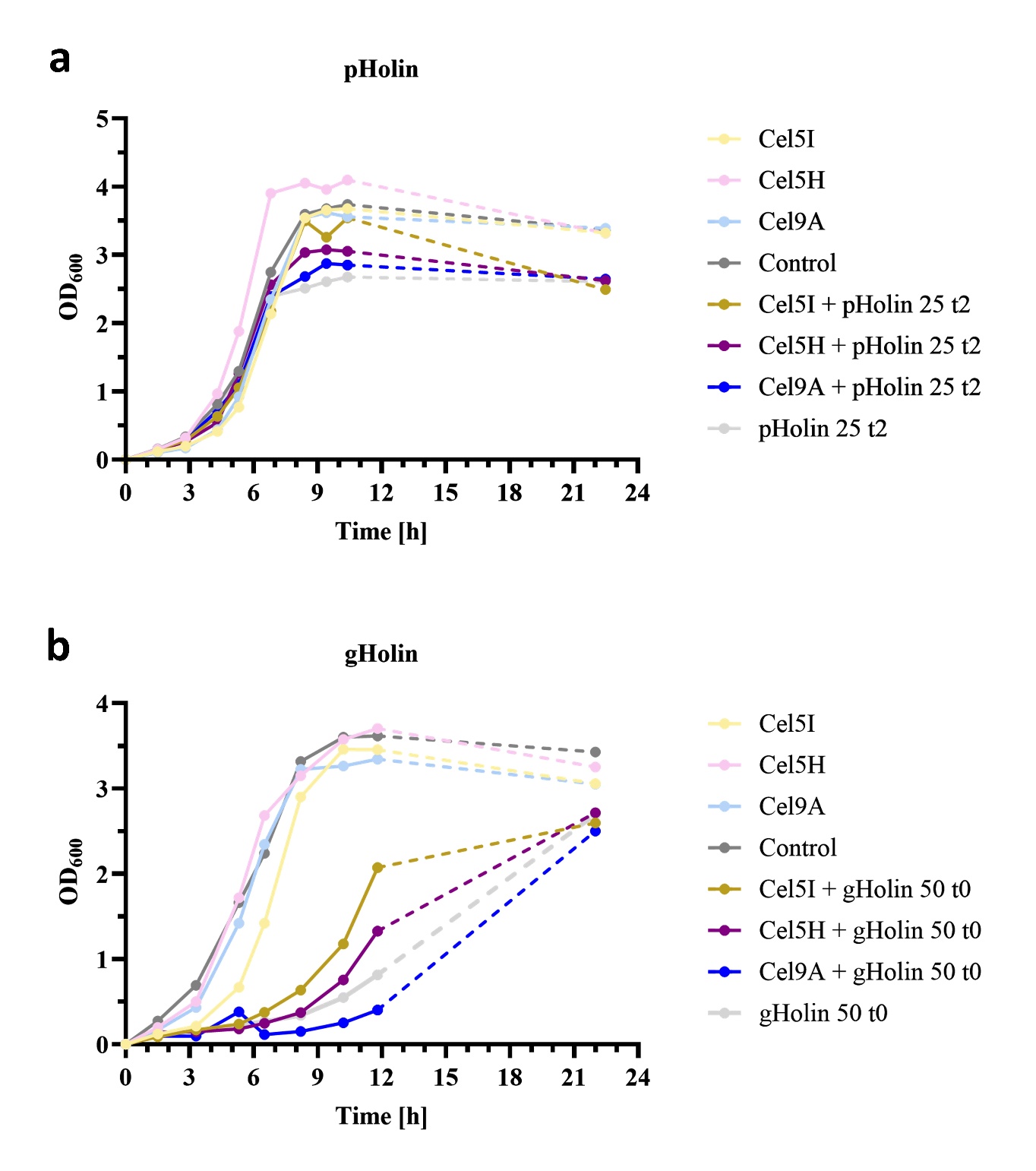


Fig. S6. Growth curves of *L. cremoris* NZ9000 strains expressing cellulases alone or with holin. Holin was expressed from the plasmid (a) or from the genome (b); in both cases under the control of nisin-inducible promoter PnisA. Cellulases Cel5I, Cel5H and Cel9A were encoded on the plasmids under the control of constitutive PepN promoter. Control: cells transformed with an empty plasmid. Expression of pHolin was induced with 25 ng/mL nisin in mid exponential growth phase (t2). Expression of gHolin was induced with 50 ng/mL nisin at the beginning of growth (t0). Cells were cultivated for approximately 22 h in M17 medium supplemented with 5 g/L glucose and 10 µg/ml of chloramphenicol. Data are presented as the mean of three biological replicates. Data points obtained over longer time interval are connected with dashed lines. This growth experiment was conducted in tubes.


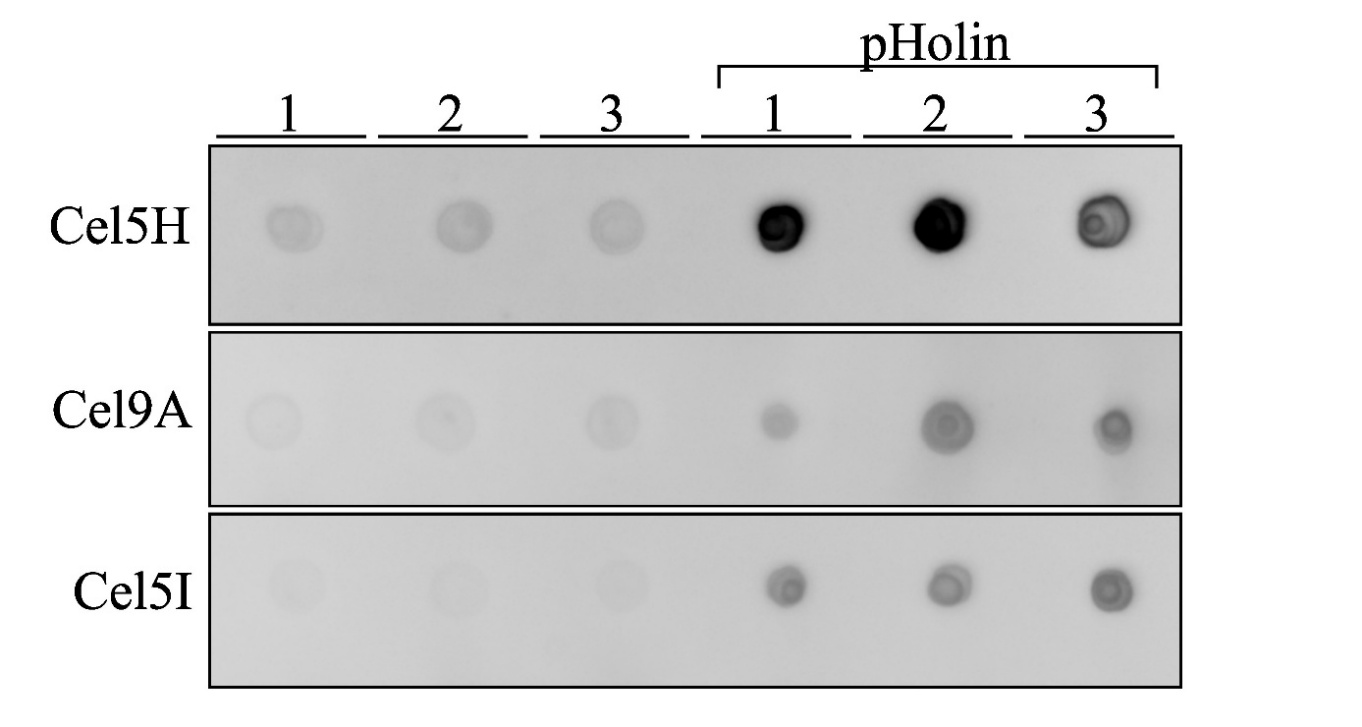


Fig. S7. Dot-blot analyses of whole intact *L. cremoris* cells secreting individual cellulase Cel5H, Cel9A and Cel5I (without the anchor domain for surface display), with or without holin. Holin expression was induced in mid exponential growth phase with 25 ng/mL of nisin. To detect cellulase, primary rabbit antibodies against flag and secondary goat anti-mouse antibodies conjugated to the fluorophore StarBright 520 were used.


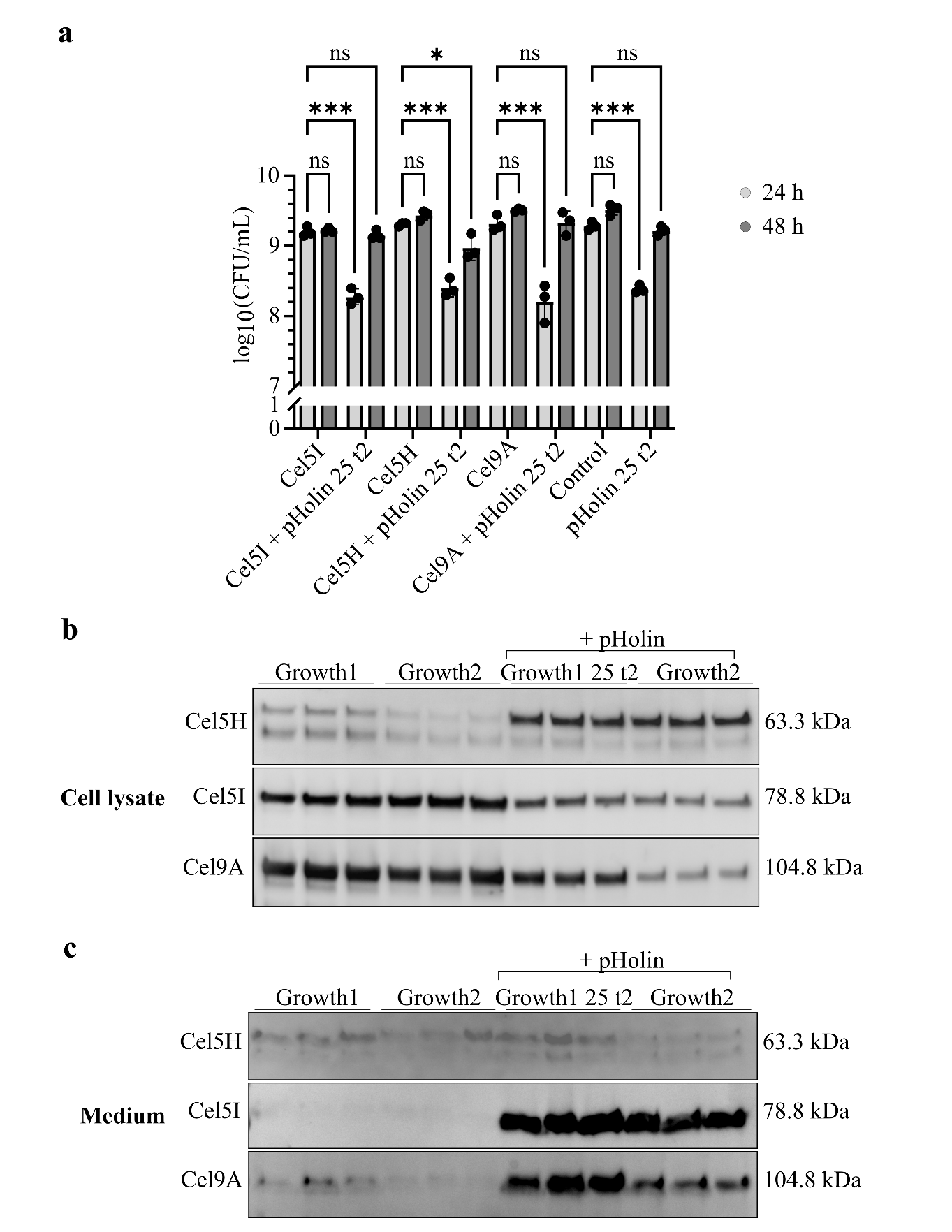


Fig. S8. Viability and cellulase yield of *L. cremoris* cells constitutively expressing cellulase (Cel5H, Cel5I or Cel9A) and inducibly expressing holin from pHolin plasmid. Growth 1: cells were grown for 20 h and holin co-expression from plasmid (+pHolin) was induced with 25 ng/mL nisin at mid exponential growth phase (t2). Growth 2: After 20 h of growth, medium was exchanged with fresh medium without nisin, and *L. cremoris* cultures expressing cellulases Cel5H, Cel9A and Cel5I were grown for another 24 h. At the end of Growth 1 and Growth 2, cell viability was evaluated with CFU/mL count (a) and the amounts of cellulases in the cell lysates (b) or in the media (c) were determined with Western blot.


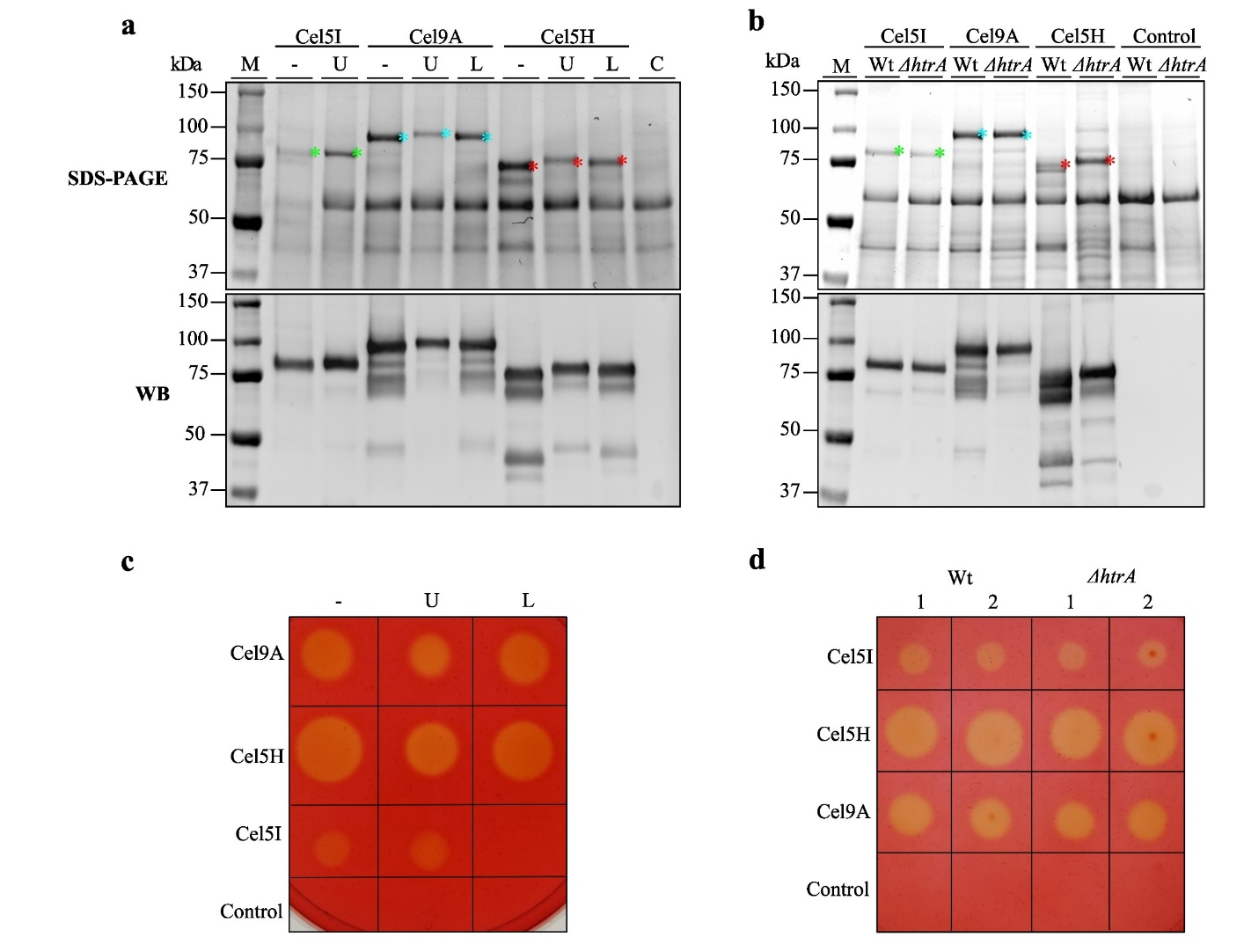


Fig. S9. Impact of N-terminus charge modification and deletion of *htrA* gene on cellulase secretion. (a) SDS-PAGE and corresponding Western blot (WB) analysis of cellulases in *L. cremoris* conditioned media. Cellulases Cel5I, Cel9A and Cel5H were expressed either without the short peptide sequence downstream of the signal sequence of the Usp45 protein (-); with the peptide corresponding to the first 15 amino acids of the mature Usp45 protein of *L. cremoris* (U); or with the peptide LEISSTCDA (L). (b) Comparison of cellulase secretion in the wild-type *L. cremoris* NZ9000 strain (Wt) and the *L. cremoris* NZ9000 strain with deleted gene for housekeeping HtrA protease (Δ*htrA*). Conditioned media of overnight cultures were analysed by SDS-PAGE and WB. All cellulases contained the N-terminal signal sequence of Usp45 protein. The green, blue and red asterisks on the SDS-PAGE indicate Cel5I, Cel9A and Cel5H, respectively. Corresponding cellulolytic activities of samples used for SDS-PAGE and WB in (a) and (b) are shown on carboxymethyl cellulose agar in (c) and (d), respectively. C or Control: *L. cremoris* strain transformed with empty pNBBX plasmid.

**References**

1. Plavec TV, Ključevšek T, Berlec A. Introduction of Modified BglBrick System in Lactococcus lactis for Straightforward Assembly of Multiple Gene Cassettes. Front Bioeng Biotechnol; 9:797521. https://doi.org/10.3389/FBIOE.2021.797521/BIBTEX

2. Štravs P, David H, Fierobe HP, Perret S, Berlec A. Development of cellulose-degrading lactic acid bacterium Lactococcus cremoris by genetic engineering. Bioresour Technol;438:133177. https://doi.org/10.1016/J.BIORTECH.2025.133177

3. Xiao YH, Pei Y. Asymmetric overlap extension PCR method for site-directed mutagenesis. Methods Mol Biol. 2011;687:277–82. doi:10.1007/978-1-60761-944-4_20.

4. Van Rooijen RJ, Gasson MJ, De Vos WM. Characterization of the Lactococcus lactis lactose operon promoter: contribution of flanking sequences and LacR repressor to promoter activity. J Bacteriol [Internet]. 1992 [cited 2024 Dec 10];174:2273. https://doi.org/10.1128/JB.174.7.2273-2280.1992

5. De Ruyter PGGA, Kuipers OP, Beerthuyzen MM, Van Alen-Boerrigter I, De Vos WM. Functional analysis of promoters in the nisin gene cluster of Lactococcus lactis. J Bacteriol;178:3434. https://doi.org/10.1128/JB.178.12.3434-3439.1996

6. Miyoshi A, Poquet I, Azevedo V, Commissaire J, Bermudez-Humaran L, Domakova E, et al. Controlled Production of Stable Heterologous Proteins in Lactococcus lactis. Appl Environ Microbiol;68:3141. https://doi.org/10.1128/AEM.68.6.3141-3146.2002

7. Debatisse K, Lopez P, Poli M, Rousseau P, Campos M, Coddeville M, et al. Redefining the bacteriophage mv4 site-specific recombination system and the sequence specificity of its attB and core-attP sites. Mol Microbiol; 2024;121:1200–16. https://doi.org/10.1111/MMI.15275
